# Cost-Effective Strategy of Enhancing Machine Learning Potentials by Transfer Learning from a Multicomponent Data Set on ænet-PyTorch

**DOI:** 10.1021/acs.jpcc.4c06235

**Published:** 2024-12-27

**Authors:** An Niza El Aisnada, Kajjana Boonpalit, Robin van der Kruit, Koen M. Draijer, Jon López-Zorrilla, Masahiro Miyauchi, Akira Yamaguchi, Nongnuch Artrith

**Affiliations:** a Department of Materials Science and Engineering, School of Materials and Chemical Technology, Tokyo Institute of Technology, 2-12-1 Ookayama, Meguro-ku, Tokyo 152-8552, Japan; b Materials Chemistry and Catalysis, Debye Institute for Nanomaterials Science, Utrecht University, Utrecht 3584 CG, The Netherlands; c Physics Department, University of the Basque Country (UPV/EHU), Leioa, Basque Country, Leioa 48940, Spain; d School of Information Science and Technology, Vidyasirimedhi Institute of Science and Technology, Rayong 21210, Thailand; e Biofunctional Catalyst Research Team, RIKEN Center for Sustainable Resource Science, 2-1 Hirosawa, Wako, Saitama 351-0198, Japan

## Abstract

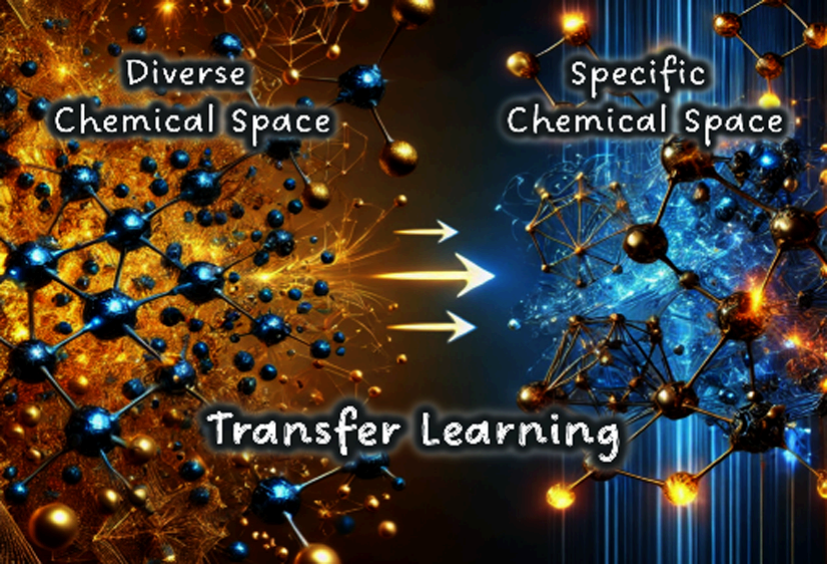

Machine learning potentials (MLPs) offer efficient and accurate material simulations, but constructing the reference ab initio database remains a significant challenge, particularly for catalyst-adsorbate systems. Training an MLP with a small data set can lead to overfitting, thus limiting its practical applications. This study explores the feasibility of developing computationally cost-effective and accurate MLPs for catalyst-adsorbate systems with a limited number of ab initio references by leveraging a transfer learning strategy from subsets of a comprehensive public database. Using the Open Catalyst Project 2020 (OC20)—a data set closely related to our system of interest—we pretrained MLP models on OC20 subsets using the ænet-PyTorch framework. We compared several strategies for database subset selection. Our findings indicate that MLPs constructed via transfer learning exhibit better generalizability than those constructed from scratch, as demonstrated by the consistency in the dynamics simulations. Remarkably, transfer learning enhances the stability and accuracy of MLPs for the CuAu/H_2_O system with approximately 600 reference data points. This approach achieved excellent extrapolation performance in molecular dynamics simulations for the larger CuAu/6H_2_O system, maintaining stable and accurate predictions for up to 250 ps, whereas MLPs without transfer learning become unstable before reaching 50 ps. We also examine the potential limitations of this strategy. This work proposes an alternative, cost-effective approach for constructing MLPs for the challenging simulation of catalytic systems. Finally, we anticipate that this methodology will pave the way for broader applications in materials science and catalysis research, facilitating more efficient and accurate simulations across various systems.

## 1 Introduction

Machine learning (ML) has rapidly advanced due to improvements in computational power, the availability of vast data sets, and algorithmic innovations. ML methods have become pivotal for material simulations in chemistry, physics, and materials science. Traditional ab initio methods, including Monte Carlo and molecular dynamics, face limitations in simulating large-scale material systems, often missing long-range interaction phenomena.^1^ Consequently, ML methods have been developed to emulate the precision of interatomic potentials derived from these accurate ab initio methods, leading to efficient ML-based interatomic potentials (MLPs).^2^

Artificial neural network (ANN) algorithms within MLP model development have gained popularity due to their capacity to discern complex relationships within various material systems by learning nonlinear patterns directly from data.^3,4^ Current advancements have further challenged the field by integrating force components into MLPs. The inclusion of force components is crucial because it enhances the accuracy and generalizability of the models, enabling better prediction of physical properties such as elastic and vibrational characteristics. This integration leads to improved stability in simulations and more efficient training processes.^5^ Despite significant progress in developing MLPs, constructing the ab initio database as the training reference remains a significant challenge, especially for complex systems like catalyst-adsorbate interactions.

One major challenge in training MLPs, especially for the catalyst-adsorbate system, is data set scarcity.^6^ Constructing a diverse custom data set for a specific chemical system requires substantial effort and resources, and training an MLP with a small data set can lead to overfitting, thus limiting its practical applications. To address this limitation, pretrained models can be fine-tuned on the target data set, resulting in more generalized MLPs than training from scratch. This approach facilitates the development of more specialized applications of MLPs.

Recent advancements have established various open-source multicomponent material databases intended for constructing machine learning potentials across diverse applications.^5,7−12^ However, due to their extensive data volumes, substantial computational resources are required to efficiently process and utilize these databases, presenting a significant challenge for many users. Therefore, we aim to investigate the feasibility of utilizing MLPs from a subset of a large multicomponent data set and applying transfer learning to construct MLPs for our material of interest.

**Figure 1:**
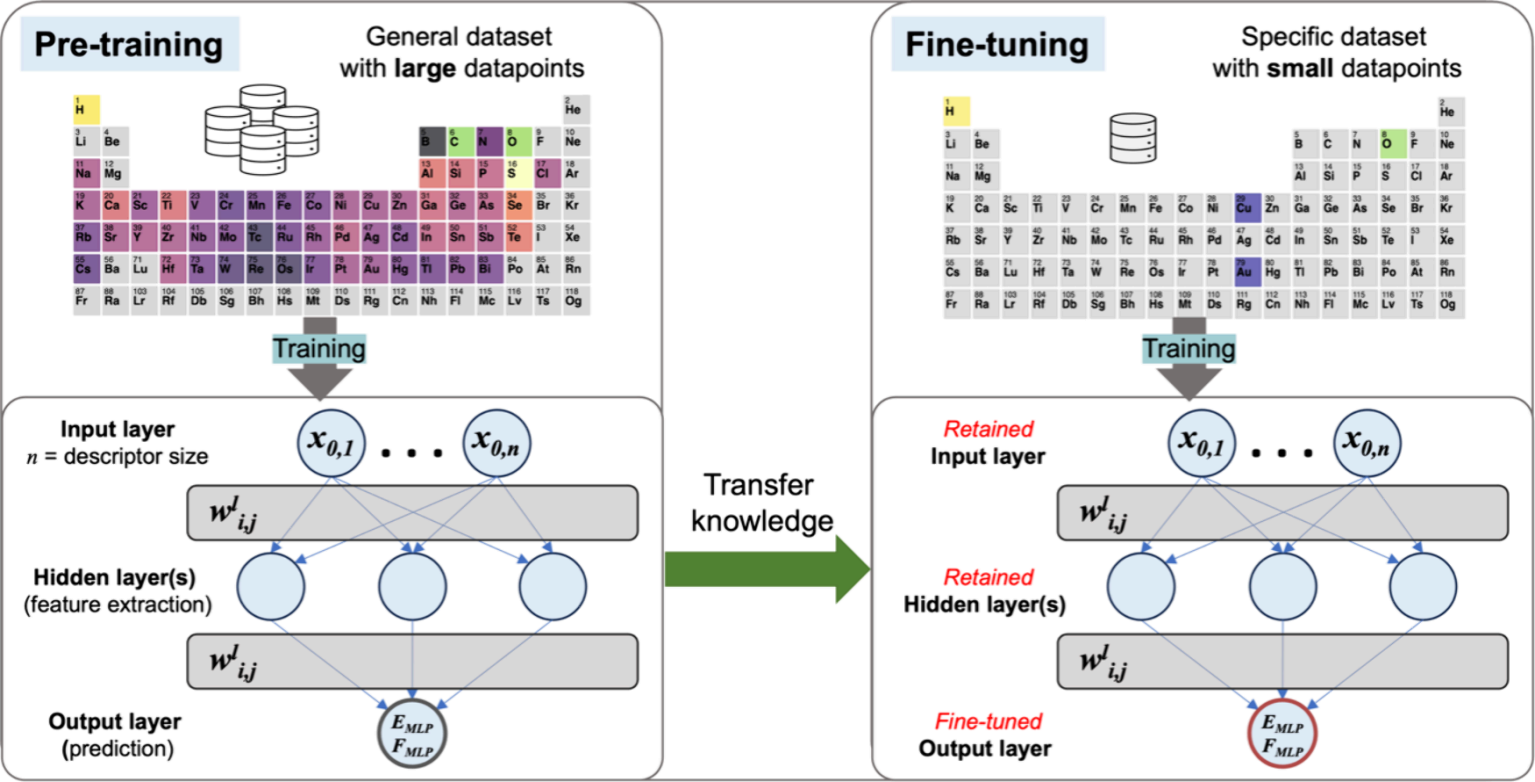
The transfer learning concept uses an ANN-based MLP model. This process is principally a knowledge transfer from pretraining to fine-tuning models. The pretraining model is trained on a large data set with vast learning information, including the atomic environment. The transfer knowledge process involves retaining the pretraining information, including the input and hidden layers. The learned information is then adapted (fine-tuned) to train a specific database to improve predictions. The process efficiently transfers knowledge from a general to a specific context, enhancing model accuracy with limited data.

Transfer learning has emerged as a powerful technique to overcome the limitations of database construction and enhance the transferability of MLPs.^13,14^ It involves training models on large, diverse data sets and then fine-tuning them on smaller, specifically targeted data sets (Figure 1). This process leverages the broad knowledge from large data sets to improve predictions on smaller ones. This approach is particularly valuable in material simulations, where obtaining large, high-quality data sets for every specific system presents a substantial challenge. Compared to routine MLP construction, which requires extensive data collection and training from scratch for each specific system, transfer learning significantly reduces the data and computational resources needed. By leveraging large, existing data sets, transfer learning accelerates development and enhances model generalization across different material systems. This efficient and cost-effective strategy is precious in fields like electrochemistry, catalysis, and materials design, where rapid and accurate simulations are crucial,^6^ and may also support quantum dynamics applications, where machine learning-derived potentials are increasingly explored.^15^

In this report, we explore cost-efficient transfer learning using subsets from the Open Catalyst 2020 (OC20) database.^16^ The database contains a comprehensive chemical environment of 2 million catalyst-adsorbate systems that are similar, though not exactly the same, as our target applications. The OC20 data set primarily includes periodic structures, while we are interested in cluster systems, which are finite and lack long-range periodicity. We employ two strategies for subset selection: (1) randomly selecting data that closely represent the original database and (2) filtering by the chemical environment close to our applications. We pretrained the MLP models using these subsets and then transferred and fine-tuned the models for our specific applications, which have smaller ab initio reference data sets. We evaluate the performance of transfer learning MLPs using molecular dynamics (MD) simulations, in comparison with MLPs constructed directly from our data set (without transfer learning). All MLP work is carried out by the Atomic Energy Network (ænet) software package.^10−12^ Specifically, we constructed the MLP using the ænet-PyTorch extension^17^ and performed the MD simulation using the ænet-LAMMPS extension.^18^ This work seeks a cost-effective and efficient way to construct MLPs for complex catalyst-adsorbate systems.

### 1.1 Machine Learning Interatomic Potential (MLP) Using an ANN Model

The success of artificial neural network (ANN)-based machine learning potentials (MLPs) largely hinges on their ability to model complex, nonlinear relationships within atomic systems.^9−11,19^ These models effectively learn intricate patterns of atomic interactions regardless of the number of atoms, making them highly accurate for predicting material properties. ANNs operate by taking input features and processing them through multiple layers of interconnected “neurons”, each applying a mathematical transformation. The output layer then delivers the final prediction.

In constructing MLPs, the ANN model interpolates atomic energy from reference ab initio calculations, such as density functional theory (DFT). Behler and Parrinello introduced a method to harness the versatility of ANNs in creating practical and reusable interatomic potentials.^4^ This approach, later expanded by Artrith et al. for multiple chemical species,^20^ involves decomposing the total energy *E*(σ) of an atomic structure (σ) into individual atomic energy contributions *E*_*i*_:
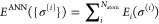
1

The key to the success of ANN-based MLPs lies in their ability to predict energies and forces for new structures regardless of the atom count, although they are confined to the chemical species present in the training data. This is accomplished by summing up the energy contributions from each atom, where each contribution *E*_*i*_ is determined by the network corresponding to its element. The assumption here is that the energy contribution of each atom *i* depends solely on its local environment, denoted as σ_*i*_.

By defining these environments through atomic fingerprints or descriptors and training on the total energy of the system, the resulting potential gains generalizability, independent of the atom count. These descriptors must meet certain symmetry conditions with respect to atom exchanges, rotations, and translations of structures, as well as the smoothness of descriptor functions. Descriptors play a critical role in ANNs by representing the atomic environment in a format that the neural network can process. They transform the atomic positions and types into numerical values that encapsulate the essential features of the system.^21,22^

### 1.2 ænet-PyTorch

ænet-PyTorch is a powerful implementation designed to train artificial neural network (ANN)-based machine learning potentials (MLPs) efficiently using the PyTorch framework.^17^ Developed as an extension of the atomic energy network (ænet), ænet-PyTorch leverages the computational power of graphics processing units (GPUs) to significantly accelerate the training process, making it feasible to include both energy and force data in the training, which enhances the accuracy and stability of the resulting MLPs.

The original ænet code,^11,12^ written in C and Fortran, supports the use of various descriptors to represent the local atomic environments. Among these, the Chebyshev descriptors introduced by Artrith et al. have shown great promise.^11^ These descriptors utilize Chebyshev polynomials to create a set of numerical values that describe the atomic environment around each atom, capturing both radial and angular dependencies. The Chebyshev descriptors are particularly efficient in handling systems with multiple chemical species because the size of the descriptor does not scale with the number of species.

The Chebyshev descriptor Τ_*n*_ for an atom *i* can be represented as
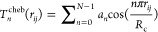
2where *r*_*ij*_ is the distance between atoms *i* and *j*, *R*_c_ is the cutoff radius, and *a*_*n*_ are the coefficients of the Chebyshev polynomials. This formulation ensures that the descriptors are continuous and differentiable, which is crucial for the stability and accuracy of the MLPs.

ænet-PyTorch integrates these descriptors into the training process, allowing the neural network to learn from both energy and force data.^17^ The training workflow involves grouping atoms of the same species and processing their descriptors in parallel, significantly speeding up the computation. By utilizing PyTorch’s built-in routines for tensor operations, the implementation achieves great scalability and performance, especially on GPUs. Using force information in the training process is essential for improving the generalizability and transferability of the MLPs. In ænet-PyTorch, the loss function combines both energy and force errors, weighted by a parameter α, allowing the model to balance the accuracy between energy and force predictions. The objective function  is defined as
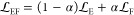
3where  and  are the root-mean-squared errors (RMSE) for energy and forces, respectively. This approach ensures that the trained MLPs can accurately predict atomic forces, which are critical for molecular dynamics simulations and other applications that require detailed atomic-level insights. By incorporating advanced descriptors like the Chebyshev polynomials and leveraging the computational power of GPUs, ænet-PyTorch facilitates the creation of highly accurate and transferable MLPs. This makes it an invaluable tool for researchers working on complex material simulations, including those involving catalyst-adsorbate interactions, as it enables the efficient and cost-effective training of machine learning potentials.

## 2 Methods

### 2.1 Selecting Subsets from the OC20 Data Set

The Open Catalyst 2020 (OC20) database is a comprehensive resource for studying catalyst-adsorbate systems, offering relaxed atomic structures and energy and force data from DFT calculations. It includes diverse catalyst structures with adsorbates containing carbon, hydrogen, nitrogen, and oxygen. For cost-effective transfer learning, we compared two subset selection strategies for pretraining models: (1) randomly selecting representative data and (2) filtering by the chemical environment relevant to our applications. Specifically, for our transfer learning application involving copper–gold alloy clusters and water (CuAu/H_2_O) systems, the second strategy focused on selecting structures containing “Cu” and “Au” to construct and utilize MLPs efficiently.

### 2.2 MLP Construction in ænet-PyTorch

**Figure 2:**
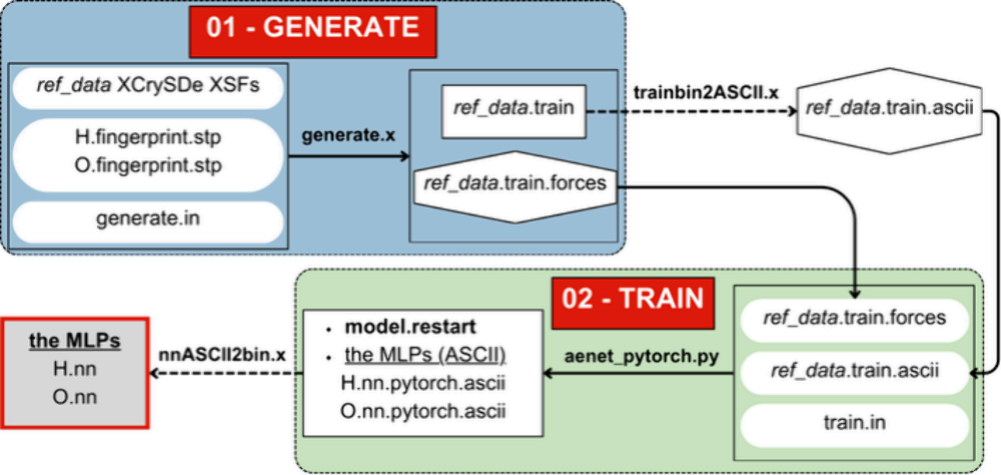
Technical workflow of constructing the machine learning potential (MLP) using ænet-PyTorch for compounds containing hydrogen (H) and oxygen (O) as an example.

In general, constructing MLP in ænet consists of two main steps: (i) generation of the training set, which processes a compilation of the references data set into a training set file; (ii) MLP model training, a process to construct the MLP models based on the generated training set file. In the ænet-PyTorch extension, the training of forces is possible. Thus, the generation of the training set will result in 2 files (energy set and forces set) if force training is enabled. Overall, the MLP construction in ænet-PyTorch is summarized in Figure 2.

#### 2.2.1 Generation of the Training Set Using generate.x and trainbin2ASCII.x Tools

In generating the training set, one should provide input files, including the structural energy and force reference data in XCrySDen Structure Format (XSF), invariant basis or structural fingerprints for each element in the training set, element.fingerprint.stp files, and principle input files of generate.in. After executing generate.x, this process will result in the training set in the binary format ref_data.train. When the force is included in the training set, this process will also result in an additional file ref_data.train.forces. Further, we convert ref_data.train into a Python-readable ASCII format (ref_data.train.ascii) training set by executing the trainbin2ASCII.x tool.

#### 2.2.2 Construction of MLP Using the ænet_pytorch.py Tool

In the routine of MLP construction using ænet, the train.x tool is used. However, in the ænet-PyTorch extension, we use the aenet_pytorch.py tool and execute it using Python. In this process, the input files for this step include the MLP training parameter in the train.in file along with ref_data.train.ascii and ref_data.train.forces from the previous step. We implement a data set split into 90% training and 10% test data during the training process. The training process results in the standard outputs, including the MLP for each element in the training set in ASCII format and model.restart as the training checkpoint. Finally, the executable binary MLPs (element.nn) for ænet-LAMMPS can be obtained by executing the nnASCII2bin.x tool.

#### 2.2.3 Hyperparameter Tuning

To achieve optimal accuracy for the MLP models, we experimented with various hyperparameters during the generation of the training set and MLP training. For the generation of the training set, we focused on optimizing the size of descriptors and the cutoff radius dimensions. This involved adjusting hyperparameters within the fingerprint setup for each element (*.stp files). We explored descriptor sizes ranging from 36 to 68 by varying the degrees of radial and angular expansion functions (“radial_N” and “angular_N”). We surveyed the optimal radial cutoff radius (“radial_Rc”) to be between 6 and 14 Å, and the angular cutoff radius (“angular_Rc”) to be between 4 and 8.

Furthermore, to select the optimal hyperparameters for training, we evaluated various options, including optimization methods (Adam, Adadelta, Adagrad, Adamax, and AdamW), learning rates (10^–6^ to 10^–2^), and weight decays (10^–5^ to 10^–2^), with a fixed batch size of 256. Potential MLP models were chosen based on both accuracy (RMSE value) and training stability to ensure effective transfer learning to different data sets.

### 2.3 Transfer Learning Interatomic Potential

**Figure 3:**
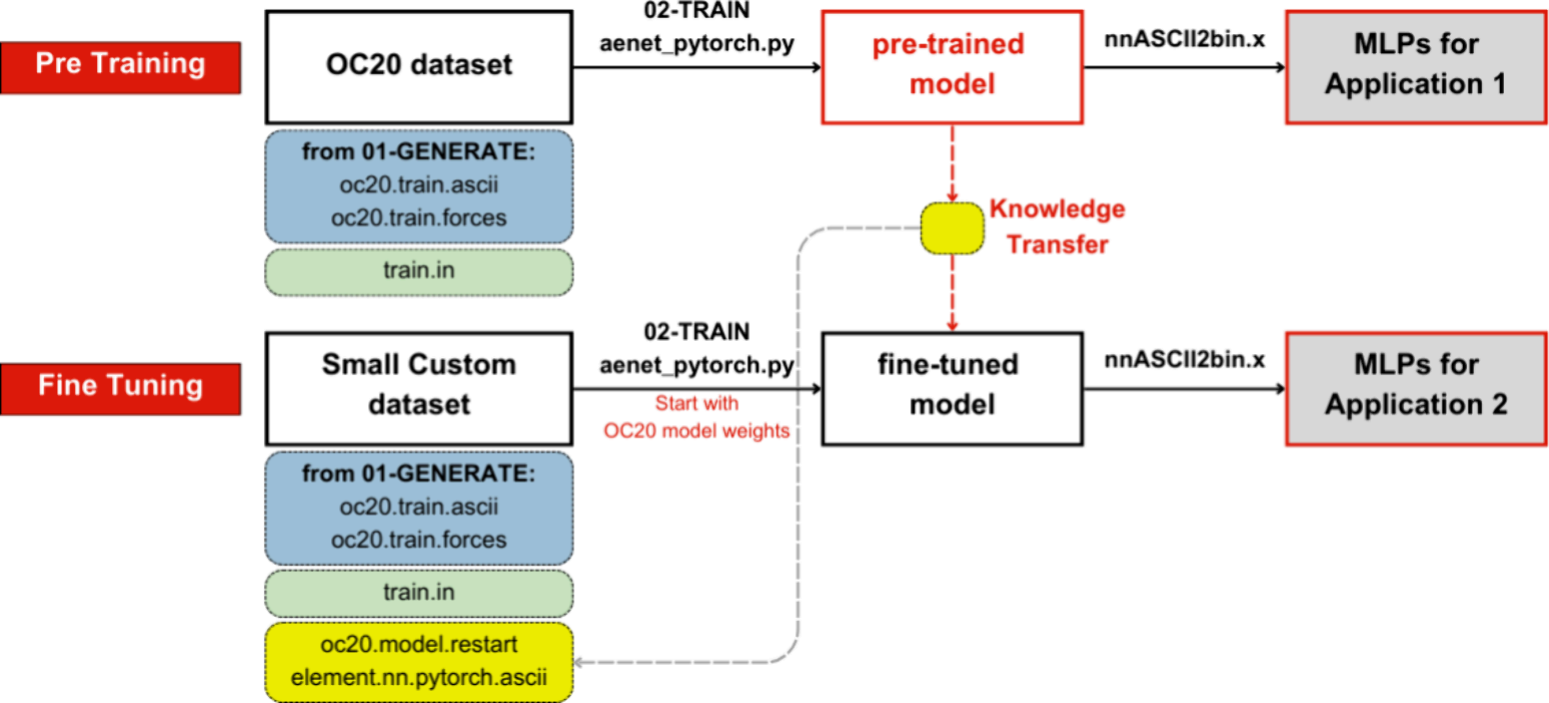
Technical workflow of transfer learning using ænet-PyTorch.

To train the MLP using the transfer learning approach, ænet-PyTorch requires the model.restart and element.nn.ascii from the source task (Figure 3), where the model was trained with a large and diverse database, in this case, the OC20 subsets. In the transfer learning process, the ænet-PyTorch framework initializes training on the target task, which involves a specific application database, with pretrained weights instead of random initialized weights. This method anticipates that the knowledge gained from the source task can significantly enhance the accuracy and generalizability of MLPs to unseen data, particularly when the training database for the target task is very small. In this case, the architecture of ANN should be fixed during the process.

From the pretrained MLP models constructed with the three OC20 subsets, we performed transfer learning to develop fine-tuned MLPs for our data set of a CuAu alloy cluster with one adsorbed water molecule (CuAu/H_2_O), using approximately 8000 DFT reference structures from the ab initio molecular dynamics simulation (AIMD) trajectory (see Supporting Information Section S1 for all AIMD details). Additionally, we tested the generalizability of the best MLP models for MD simulations of a larger CuAu alloy cluster with 6 water molecules (CuAu/6H_2_O).

## 3 Results and Discussion

### 3.1 The Selected OC20 Subsets

**Figure 4:**
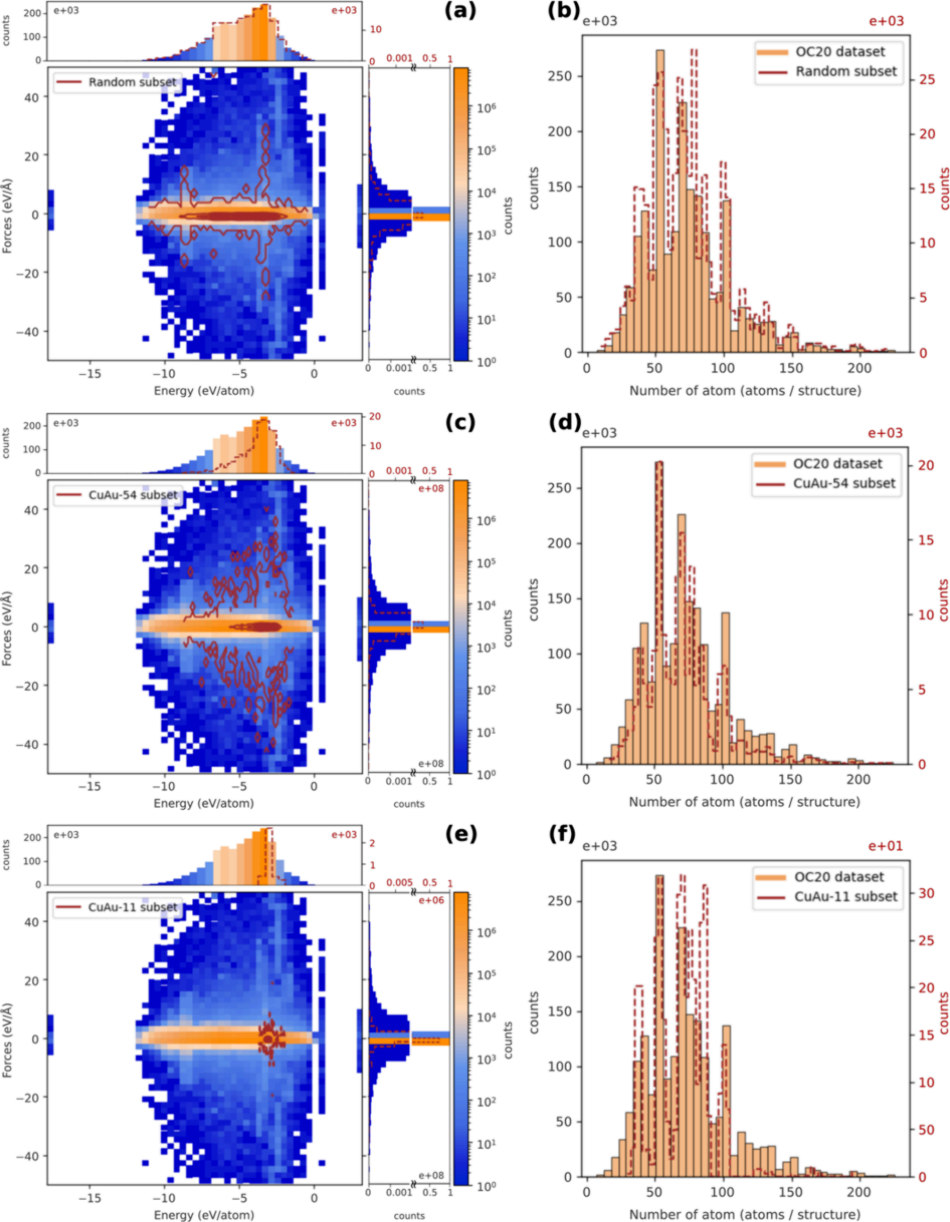
Energy–force 2D histogram of the OC20 data set overlaid by the (a) random, (c) CuAu-54, and (e) CuAu-11 subsets. Distribution of the number of atoms per structure of the OC20 data set overlaid by the (b) random, (d) CuAu-54, and (f) CuAu-11 subsets.

Figure 4a,c,e presents the 2D histogram distribution of energy and forces for each OC20 subset in Table 1, overlaid with the original OC20 data set distribution. Figure 4b,d,f shows the distribution of the number of atoms per structure for each subset. Additionally, Figure S3b–d illustrates the element distributions for the elements included in each subset. The original data set contains 56 elements (Figure S3a), with energy and force values concentrated around −4 eV/atom and 0 eV/Å, respectively. The data set includes structures with atom counts ranging from about 2 to over 200 atoms per structure, with most samples containing 50–100 atoms per structure.

**Table 1:** Subset of OC20 for the Pretrained MLP Models

OC20 subset	data points	selection process
random	200K	based on the closest representation of data sets in terms of energy, forces, number of atoms/structures, and element distribution
CuAu-54	170K	filter any structures that contain at least “Cu”, “Au”, and “Cu + Au”
CuAu-11	4K	filter any structures that contain at least “Cu + Au”

For the random subset, we aimed to randomly select 10% of the OC20 data set to closely represent the original data set. However, balancing the included elements, the energy–force distribution, and the number of atoms per structure proved challenging. As a result, we prioritized the distribution of energy over forces, given that force data are three times more prevalent. Ultimately, the random subset contains 56 elements with a distribution similar to the original data set, depicting energy and force ranges from 0 to −12 eV/atom and −30 to 30 eV/Å, respectively.

The next two subsets were selected based on structures containing “Cu” and/or “Au”. We were not concerned about the adsorbates since hydrogen and oxygen are dominant within the database (Figure S3a). First, we filtered structures that contain “Cu”, “Au”, and “Cu” + “Au”, resulting in the CuAu-54 subset, which contains approximately 170,000 structures and 54 elements (Figure S3c). Compared to the random subset, the CuAu-54 subset has a narrower energy distribution but a wider range of force distribution.

The final subset, named CuAu-11, was selected based on its similarity to our target material CuAu/H_2_O. This subset contains structures with only a combination of “Cu” + “Au” in a structure, resulting in 11 elements and a total of 4000 structures. The energy and force values are concentrated around −3 eV/atom and 0 eV/Å, respectively. The structures in the CuAu-11 subset range from 20 to 100 atoms per structure.

### 3.2 Construction of the Training Sets

#### 3.2.1 The Descriptor Dimension of the Training Data Set

**Figure 5:**
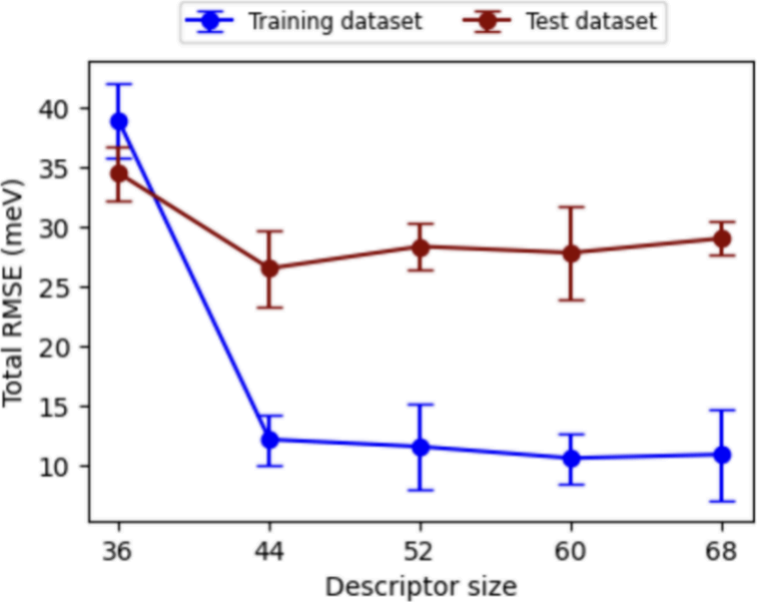
Accuracy of MLPs of the given subsets as a function of the descriptor dimension that represents the local atomic environment.

Varying the descriptor dimension is crucial for optimizing computational cost, feature representation, efficiency, and generalization.^20,23,24^ This experiment aims to find the “sweet spot” for the model’s effectiveness in predicting the properties of new atomic environments. Figure 5 shows the RMSE of the MLP model as a function of descriptor size, measured from independent models from the given subsets. The RMSE decreases significantly as the descriptor size increases from 36 to 44, indicating improved predictive accuracy. Beyond 44, further improvements are marginal. Thus, we selected a descriptor size of 44 as the optimal balance between computational efficiency and predictive performance. These results align with previous findings on computational tractability and multispecies interaction capture.^20^ This descriptor size adequately characterizes the local atomic environment, ensuring accurate MLP predictions without unnecessary computational costs.

#### 3.2.2 The Cutoff Radius Dimension of the Training Data Set

The setup of radial and angular cutoffs significantly impacts the computational efficiency and accuracy of simulations. Learning from various practices using different descriptors that contain RDF and ADF components, the cutoff radius is set based on the type of interaction suitable for specific systems.^11,25,26^ The radial cutoff determines the maximum distance within which atomic interactions are considered, which is essential for capturing relevant interactions without unnecessary computations that could slow down the simulation. For short-range interactions (typically 6–10 Å), this cutoff ensures computational efficiency and accuracy for small molecules and simple systems where long-range effects are minimal. Medium-range interactions (typically 10–12 Å) offer a balance between accuracy and computational cost, making them suitable for a broad range of materials science applications. For long-range interactions (typically 12–15 Å), the radial cutoff is extended to capture significant interactions in large biomolecules and complex materials, ensuring the model’s accuracy in these scenarios.

The angular cutoff defines the level of detail in the angular dependencies of atomic interactions. Low-order angular cutoffs (typically 3–4) are appropriate for simple systems where detailed angular dependencies are not critical, providing basic interaction information with minimal computational cost. Medium-order cutoffs (typically 4–6) strike a balance for most materials science and chemistry applications, capturing essential angular information without excessive computational overhead. High-order angular cutoffs (typically 6–8) are vital for highly complex systems, providing detailed angular interaction information necessary for accurately modeling anisotropic materials and surface science studies.

**Figure 6:**
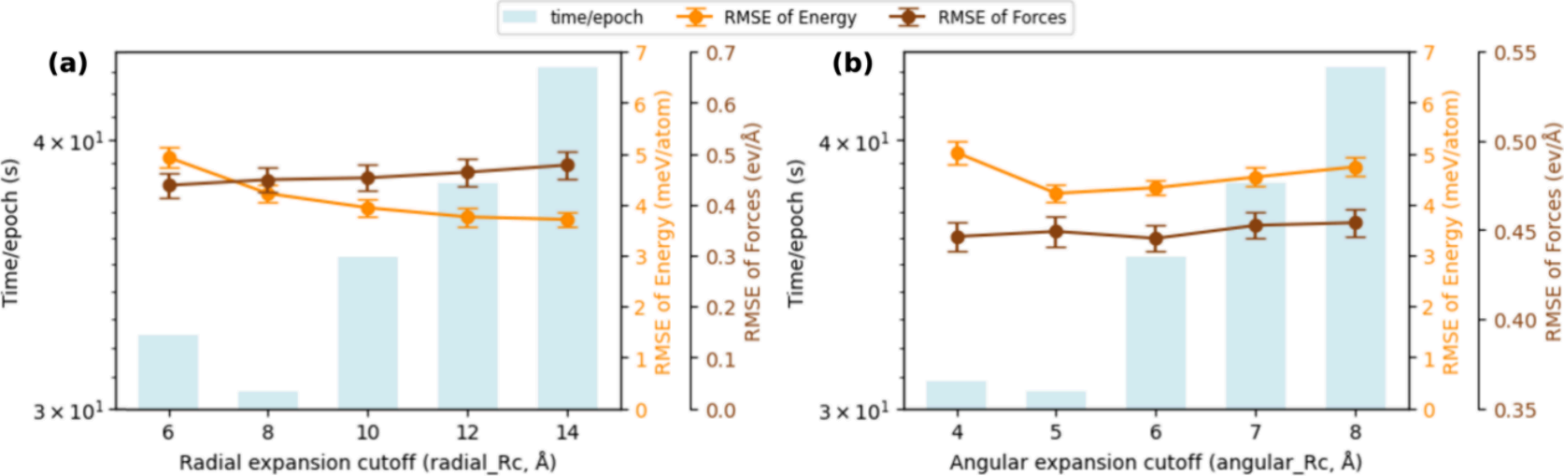
Accuracy of the MLP model as a function of the descriptor dimension that represents the local atomic environment, along with the impact on computational time. Time per epoch (blue bars, left *y*-axis) and RMSE for energy (orange line) and forces (brown line) (right *y*-axis) are shown for (a) radial cutoff (Rc) distance and (b) angular cutoff (Rc) order.

Here, we experiment with the cutoff radius for low to medium-range applications using the given training data set. Figure 6 illustrates the time per epoch and the RMSE values for energy and forces as functions of the radial and angular cutoffs. Here, an epoch represents one complete iteration through the training data set. We observed that smaller or too-small cutoff values sometimes lead to longer computation times per epoch due to the higher load needed to account for all interactions within the restricted radius. We found that a radial cutoff of 8 Å and an angular cutoff of 5 is optimal, considering both computational efficiency and accuracy for the given OC20 subsets.

### 3.3 Pretraining Interatomic Potential Based on the OC20 Subsets

#### 3.3.1 Training of Energy

While constructing the pretraining models, we observed that large learning rates (LR) often led to an unstable training process across most optimizers (Figures S6–S10). Optimizers such as Adam, Adadelta, and Adamax exhibited slow reductions in RMSE for both energy and force predictions, even when higher learning rates were applied. These Adam-based optimizers also showed varying reactions to changes in weight decay. Notably, AdamW and Adagrad were more efficient in handling both learning rate and weight decay simultaneously, providing better stability and performance in the training process.^27^ Inspecting the learning processes is necessary, particularly for transfer learning purposes, because it ensures that the pretrained model adapts well to the new, specific data set. This inspection helps identify optimal hyperparameter settings that stabilize the training process and improve model performance. The careful monitoring can also reveal how well the model retains helpful knowledge from the pretrained phase while adjusting to new data, ultimately enhancing the accuracy and reliability of the transfer learning application.

**Figure 7:**
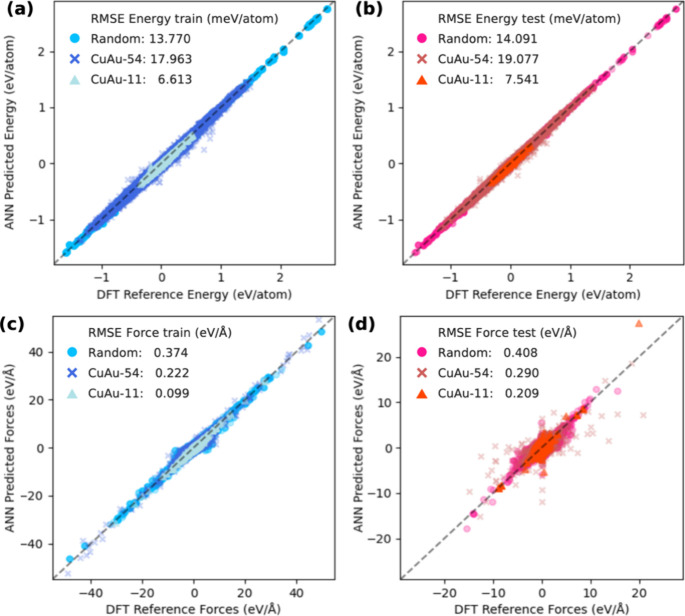
Evaluation of pretraining MLP models’ prediction for each OC20 subset: random, CuAu-54, and CuAu-11. (a) and (b) show the energy predictions, while (c) and (d) illustrate the force predictions. Blue markers represent training data, and red markers represent test data.

Figure 7 displays the energy and force prediction power of the best MLP fitting results for each OC20 subset. Figure 7a,b shows the energy predictions, while Figure 7c,d illustrates the force predictions. In the energy prediction (Figure 7a,b), the results indicate that the CuAu-11 subset has the lowest RMSE for training and test data, suggesting that it has the most accurate energy predictions among the subsets. For the training data (Figure 7a), the random subset achieves an RMSE of 13.770 meV/atom, the CuAu-54 subset an RMSE of 17.963 meV/atom, and the CuAu-11 subset an RMSE of 6.613 meV/atom. For the test data (Figure 7b), the random subset achieves an RMSE of 14.091 meV/atom, the CuAu-54 subset an RMSE of 19.077 meV/atom, and the CuAu-11 subset an RMSE of 7.541 meV/atom. We expect that these results arise from the nature of energy and force distribution within the subset.

#### 3.3.2 Training of Forces

Prior research indicates that including at least 10% of forces in the training set is adequate.^17^ In the original article of ænet-PyTorch, López-Zorilla et al. discuss the balance between energy and force information in training data. It is emphasized that while all structures contribute to energy fitting, only a subset is used for force training. An experiment removing energy-only structures from training showed a disproportionate impact on energy prediction accuracy, mainly when data coverage is sparse. Force predictions were less affected. This implies that while force data enhance the model around known structures, predictions for configurations far from the training set are less reliable.

**Figure 8:**
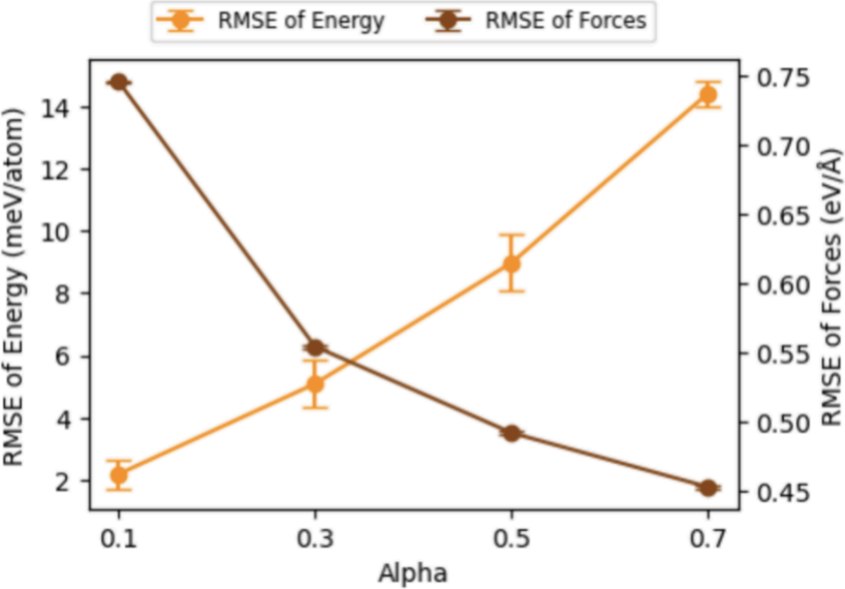
Accuracy of energy and force prediction as the function of alpha (α).

Besides the hyperparameter mentioned in the energy training section, one hyperparameter that greatly influences the force prediction accuracy is alpha (α) since this parameter balances energy and force components in the combined loss function during training. As α increases, the model prioritizes minimizing force loss, leading to better force prediction accuracy but worse energy prediction accuracy. This trade-off occurs because higher α shifts the model’s focus and capacity toward fitting forces more precisely, potentially at the expense of energy predictions. Our results in Figure 8 and Figure S5 show that while increasing α improves force RMSE, it simultaneously raises the energy RMSE, indicating that the model’s ability to predict energy diminishes as it prioritizes force accuracy.

Further, in the previous conclusion based on the TiO_2_ database,^17^ the α values of 0.1–0.3 can be optimal for general application. However, we found that α = 0.1 leads to a loose force prediction in our training data set (Figure S5). The α of 0.3–0.5 might be suitable for the OC20 subsets. This shift can be attributed to the increased complexity and diversity of the OC20 data set, which includes a wider variety of atomic environments and interactions. In such a data set, a higher α is necessary to ensure the model allocates sufficient capacity to capture the more nuanced and varied force interactions accurately. As a result, the model can better generalize across the diverse configurations present in OC20, leading to improved overall performance, especially in energy predictions. This indicates that the balance between energy and force accuracy, governed by α, is data set-dependent and needs to be carefully tuned to match the specific requirements and characteristics of the training data.

Overall, similar to the energy predictions, the CuAu-11 subset demonstrates the lowest RMSE for both training and test data in force predictions. However, the MLP model from the CuAu-54 subset has better predictability than the MLP model from the random subset. For the training data (Figure 7c), the random subset achieves an RMSE of 0.374 eV/Å, the CuAu-54 subset an RMSE of 0.222 eV/Å, and the CuAu-11 subset an RMSE of 0.099 eV/Å. For the test data (Figure 7d), the random subset achieves an RMSE of 0.408 eV/Å, the CuAu-54 subset an RMSE of 0.290 eV/Å, and the CuAu-11 subset an RMSE of 0.209 eV/Å.

### 3.4 Transfer Learning Interatomic Potential to the CuAu/H_2_O Data Set

#### 3.4.1 Trial on the Direct Application

Before applying transfer learning, we tested our hypothesis by evaluating MLPs trained on OC20 (our pretrained models) on two independent test sets to predict adsorption energies: Pd–Au/H_2_/O_2_^28^ and Cu–Au–Pt/O_2_. Both systems exhibited significant discrepancies between the adsorption energies predicted by the pretrained MLP models and the reference DFT values. The results from these tests are detailed in the Supporting Information sections S2–S4. As expected, these tests confirmed that the pretrained models cannot be used directly for our complex catalyst models.

#### 3.4.2 Accuracy Comparison of Transfer Learning from Different Subsets

**Figure 9:**
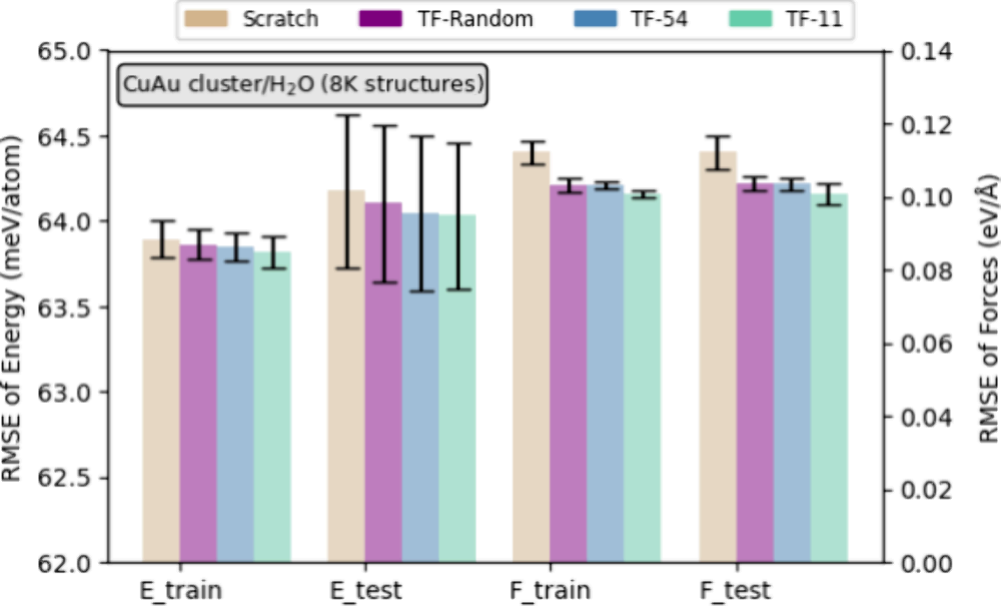
Energy and force prediction accuracy of MLPs for CuAu/H_2_O constructed from scratch and using transfer learning from OC20 subsets: random (TF-Random), CuAu-54 (TF-54), and CuAu-11 (TF-11).

Herein, we highlight the benefits of using the OC20 pretrained model for transfer learning. Figure 9 and Table S3 show the energy and force prediction accuracy of the MLPs for CuAu/H_2_O, constructed from scratch and using transfer learning from pretrained MLPs on OC20 subsets. The subsets used for transfer learning include random (TF-Random), CuAu-54 (TF-54), and CuAu-11 (TF-11). The performance is measured in terms of the RMSE for both energy and forces. The bar plot illustrates the RMSE values for both training (*E*_train_ and *F*_train_) and test (*E*_test_ and *F*_test_) data sets.

For the energy predictions, the transfer learning models exhibit consistently lower RMSE values than the model trained from scratch. Specifically, the scratch model’s energy RMSE values are 63.89 meV/atom for training and 64.18 meV/atom for testing. In comparison, the TF-11 model achieves the lowest RMSE with 63.82 meV/atom for training and 64.03 meV/atom for testing, marking a slight yet consistent improvement. Although the difference in energy RMSE is modest, this trend across both training and testing suggests that even small improvements in RMSE from transfer learning contribute to the stability of the predictions. Notably, the TF-Random and TF-54 models also demonstrate lower RMSE than the scratch model, further supporting the effectiveness of pretraining.

For force predictions, the benefits of transfer learning are even more pronounced. The scratch model’s RMSE for forces is 112.13 meV/Å for training and 112.15 meV/Å for testing. In contrast, the TF-11 model significantly reduces the RMSE to 100.72 meV/Å for training and 100.75 meV/Å for testing, representing an improvement of approximately 10.2% in force prediction accuracy. The TF-54 and TF-Random models also show reductions in force RMSE compared to the scratch model, with the TF-54 model achieving RMSE values of 103.15 meV/Å for training and 103.48 meV/Å for testing. These results clearly demonstrate that transfer learning, especially from subsets closely related to the target system, can substantially enhance force prediction accuracy.

Overall, these results clearly demonstrate that transfer learning from OC20 pretrained models significantly enhances the accuracy of energy and force predictions for the CuAu/H_2_O system. Among the subsets, the CuAu-11 subset offers the greatest benefit, likely due to its specific chemical relevance to the target system, CuAu/H_2_O. This result underscores the importance of selecting appropriate pretrained data sets for transfer learning to achieve optimal performance in machine learning potentials.

#### 3.4.3 Application for MD Simulation

The superior generalizability of the transfer learning (TF) model is evident through molecular dynamics (MD) simulations. Here, we used 574 structures of CuAu/H_2_O instead of all 8000 structures to construct the transfer learning MLPs. We then compared the performance of MLPs derived from different training approaches: training from scratch (S model) and transfer learning (TF model). The MLP training results, shown in Table 2, demonstrate that both MLP models achieved the same level of accuracy.

**Table 2:** Comparison of the RMSE in MLP Training Using Different Approaches on the CuAu/H_2_O Data Set

**approach**	***E***_**train**_**(meV)**	***E***_**test**_**(meV)**	***F***_**train**_**(meV)**	***F***_**test**_**(meV)**
direct learning on the CuAu/H_2_O data set (S model)	86.373	92.709	109.107	114.361
transfer learning from OC20→CuAu/H_2_O (TF model)	86.312	92.449	112.721	115.499

**Figure 10:**
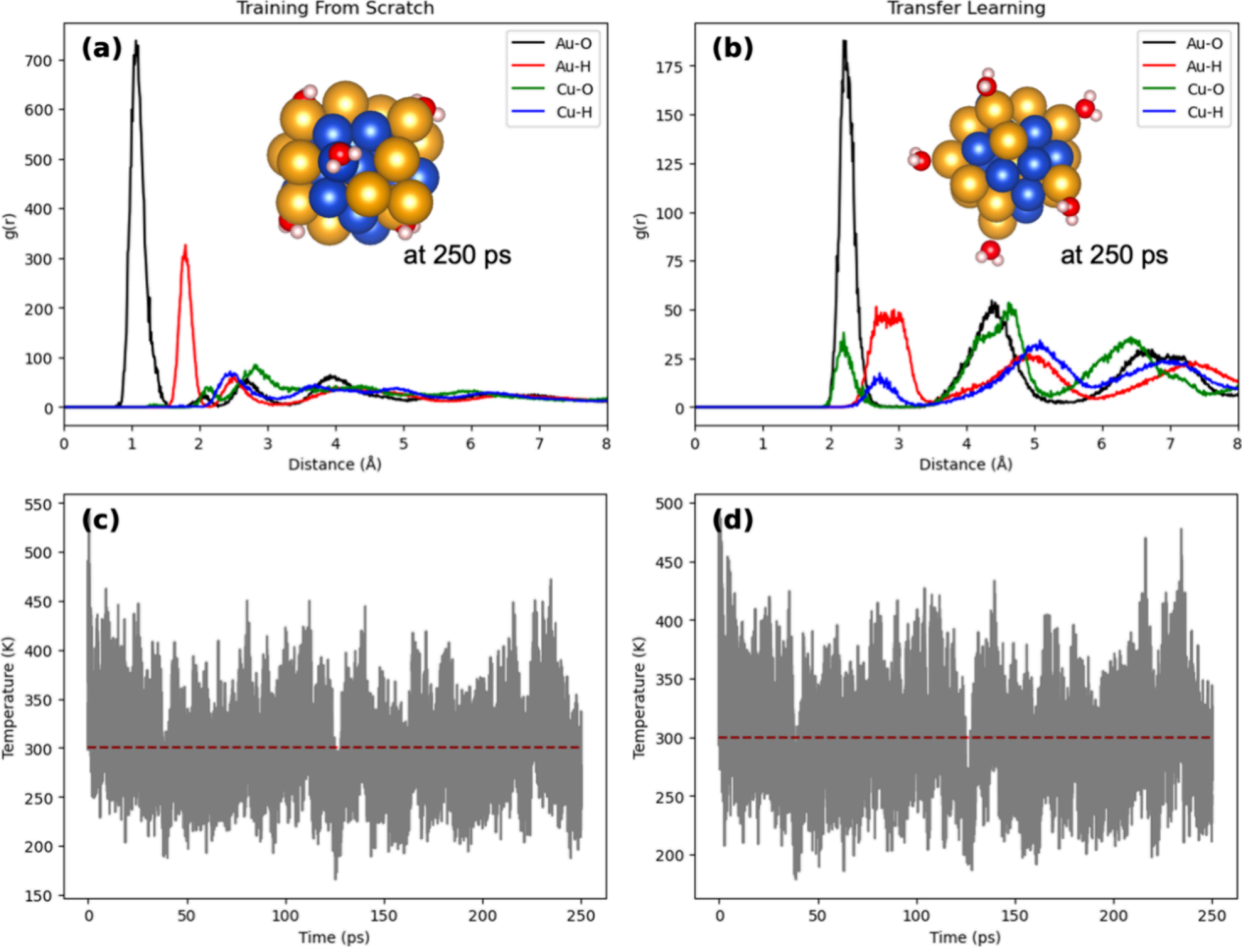
(a,b) Radial distribution function (RDF) and MD snapshot at 250 ps. (c,d) Temperature during MD simulations derived from S and TF models.

Further, to assess the extrapolation performance, we conducted 250 ps MLP-MD simulations of a CuAu/6H_2_O system using both MLPs. The simulations were performed under the canonical ensemble (NVT) using a Bussi thermostat with time steps of 0.5 fs at the temperature of 300 K. The radial distribution function (RDF) derived from the S model’s trajectory (Figure 10a) exhibits unexpectedly short Au–O distances, with the RDF of Au–O peaking at 1.065 Å. This distance is considered abnormal because it deviates significantly from the expected Au–O bond length of around 2.0–2.2 Å, commonly observed in stable systems. In contrast, the TF model shows a more realistic peak at 2.195 Å. Furthermore, both MLPs indicate Cu–O distances for adsorbed water in the range of ∼2.1–2.2 Å, yet the S model’s simulation reveals some Cu–O distances shorter than 1.5 Å, a phenomenon not observed with the TF model. These results support that the prior knowledge from the OC20 pretrained model is conserved and effectively transferred to enhance our specific task, even though this improvement is not reflected by RMSE in MLP training.

Moreover, we noted that the OC20 pretrained model cannot be directly applied to run MD simulations of CuAu/6H_2_O (Figure S4) due to the absence of metal cluster data in the OC20 database. Therefore, transfer learning becomes crucial for leveraging large chemical databases for specific chemical systems and applications of interest.

#### 3.4.4 Influence of Fine-Tuning the Data Size on Transfer Learning Accuracy

The result above shows that transfer learning can enhance MLP stability during the MD application, even though the RMSE did not indicate significant improvement. To further investigate this, we assess the effect of the fine-tuning data set size on the prediction accuracy of transfer learning. This is achieved by comparing the RMSE of the TF model to the RMSE of the S model with two different fine-tuning data set sizes: 8K and 0.5K. Here, 8K refers to transfer learning conducted with a fine-tuning data set of all 8000 structures from our CuAu/H_2_O data set, while 0.5K indicates a data set of 574 structures used for the application described in Section 3.4.3.

Given that the RMSE scale differs for the two data set sizes (8K and 0.5K), we apply a paired *t* test to evaluate the statistical significance of the differences between the TF and S models’ results. The *t* test *p*-value helps determine whether the observed differences are likely due to random chance or if they indicate a genuine effect of the data set size on prediction accuracy.^29^ A *p*-value below the significance threshold (0.05) suggests that the observed difference is statistically significant, meaning it is unlikely to be due to random chance and instead reflects a real effect of the data set size on model performance. Conversely, a *p*-value above 0.05 indicates that the observed difference is not statistically significant, suggesting it could reasonably be due to random variation rather than an actual effect of data set size.

**Figure 11:**
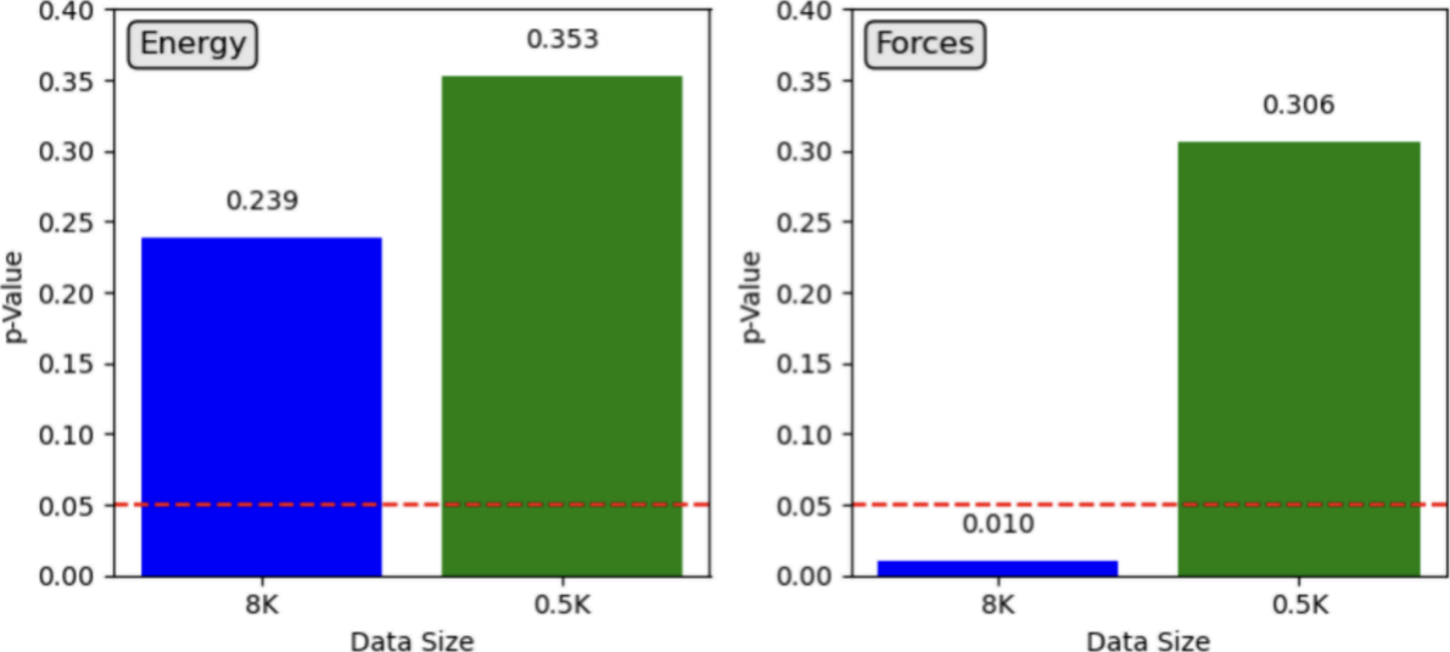
*p*-Values for (a) energy and (b) force prediction accuracy across different fine-tuning data sizes of CuAu/H_2_O.

The visualized *p*-values in Figure 11 indicate the impact of data size on the prediction accuracy of energy and forces when comparing the TF model and S model. For energy predictions, the *p*-values for the 8K (0.239) and 0.5K (0.353) data sizes are above the significance threshold of 0.05.^29^ This suggests that the differences in prediction accuracy between the TF and S methods for energy are not significant, regardless of the data size. Thus, data size does not remarkably appear to affect the prediction accuracy for energy.

In contrast, the *p*-values for the force component show a different trend. The *p*-value for the 8K data size (0.010) is below the significance threshold, indicating a statistically significant difference in force prediction accuracy between the TF (transfer learning) and S (training from scratch) methods. On the other hand, the *p*-value for the 0.5K data size (0.306) is above the threshold, suggesting no significant difference in prediction accuracy at this smaller data size. This implies that a larger data set (8K) is necessary to detect meaningful differences in the prediction accuracy of forces between the TF and S conditions, highlighting the impact of data size on the reliability of force predictions.

These results indicate that transfer learning’s benefits become more pronounced with larger data sets. While the RMSE values for the smaller data set do not show a significant advantage for the TF model, the MD simulations reveal its superior stability and physical accuracy. This highlights that RMSE alone does not always indicate model performance, especially in dynamic simulations. Transfer learning, therefore, proves crucial for leveraging large chemical databases to enhance specific tasks, ensuring better generalization and stability in practical applications.

## 4 Conclusions

We aimed to explore a cost-effective approach for constructing machine learning potentials (MLPs) for challenging catalyst-adsorbate systems using transfer learning. By leveraging an available public data set and carefully selecting appropriate subsets, we demonstrated two strategies: random selection of subsets and specific selection of subsets. Our findings indicate that specific subset selection, based on chemical environment similarity, results in better accuracy, underscoring the importance of data set relevance in enhancing MLP transfer learning. We further investigated the feasibility of transferring the MLP model to a smaller fine-tuning data set with ∼600 data points. While the RMSE values for energy and force predictions did not show significant improvement, the generalizability of the transfer learning MLP was evident through stable MD simulation results. The MLP derived from transfer learning proved to be more stable and accurate for long-term MD simulations, whereas the MLP constructed from scratch did not achieve comparable stability. Our results also highlight the advantage of using a larger fine-tuning reference data set to significantly enhance the performance and applicability of the MLP, making it more robust for practical applications. Further, we learned that evaluating MLPs based solely on RMSE values is insufficient. Applying the MLPs in practical simulations is crucial to ensure their usefulness and effectiveness. Overall, this study underscores the effectiveness of transfer learning in constructing accurate and stable MLPs for complex material systems, emphasizing the importance of data set selection, size, and practical application in optimizing model performance.

## Data Availability

The data from the density functional theory (DFT) calculations can be obtained from the GitHub repository at https://github.com/atomisticnet/transfer-learning/tree/main/OC20.

## Supporting Information Available

Methods for CuAu/H_2_O and Cu–Au–Pt/O_2_ data set generation, results for a direct application of the pretraining MLP model on Cu–Au–Pt/O_2_ and H_2_/O_2_ adsorption on a Pd–Au alloy, data analysis for the element distribution of the OC20 data set and the selected subsets, hyperparameter tuning for the MLP model training, and direct application of the pretrained MLP model for the MD simulations of CuAu/6H_2_O (PDF)

## References

[ref1] Anstine D. M.; Isayev O. Machine Learning Interatomic Potentials and Long-Range Physics. J. Phys. Chem. A 2023, 127, 2417–2431. 10.1021/acs.jpca.2c06778. 36802360 PMC10041642

[ref2] Mueller T.; Hernandez A.; Wang C. Machine learning for interatomic potential models. J. Chem. Phys. 2020, 152, 050902 10.1063/1.5126336. 32035452

[ref3] Lorenz S.; Scheffler M.; Gross A. Descriptions of surface chemical reactions using a neural network representation of the potential-energy surface. Phys. Rev. B: Condens. Matter Mater. Phys. 2006, 73, 115431 10.1103/PhysRevB.73.115431.

[ref4] Behler J.; Parrinello M. Generalized neural-network representation of high-dimensional potential-energy surfaces. Phys. Rev. Lett. 2007, 98, 146401 10.1103/PhysRevLett.98.146401. 17501293

[ref5] Chen C.; Ong S. P. A Universal Graph Deep Learning Interatomic Potential for the Periodic Table. Nat. Comput. Sci. 2022, 2, 718–728. 10.1038/s43588-022-00349-3. 38177366

[ref6] Artrith N. Learning What Makes Catalysts Good. Matter 2020, 3, 985–986. 10.1016/j.matt.2020.09.012.

[ref7] Lee K.; Yoo D.; Jeong W.; Han S. SIMPLE-NN: An efficient package for training and executing neural-network interatomic potentials. Comput. Phys. Commun. 2019, 242, 95–103. 10.1016/j.cpc.2019.04.014.

[ref8] Lysogorskiy Y.; et al. Performant implementation of the atomic cluster expansion (PACE) and application to copper and silicon. NPJ Comput. Mater. 2021, 7, 97 10.1038/s41524-021-00559-9.

[ref9] Wang H.; Zhang L.; Han J. DeePMD-kit: A deep learning package for many-body potential energy representation and molecular dynamics. Comput. Phys. Commun. 2018, 228, 178–184. 10.1016/j.cpc.2018.03.016.

[ref10] Cooper A. M.; Kästner J.; Urban A.; Artrith N. Efficient training of ANN potentials by including atomic forces via Taylor expansion and application to water and a transition-metal oxide. NPJ Comput. Mater. 2020, 6, 54 10.1038/s41524-020-0323-8.

[ref11] Artrith N.; Urban A.; Ceder G. Efficient and accurate machine-learning interpolation of atomic energies in compositions with many species. Phys. Rev. B 2017, 96, 014112 10.1103/PhysRevB.96.014112.

[ref12] Artrith N.; Urban A. An implementation of artificial neural-network potentials for atomistic materials simulations: Performance for TiO_2_. Comput. Mater. Sci. 2016, 114, 135–150. 10.1016/j.commatsci.2015.11.047.

[ref13] Varughese B.; et al. Active and Transfer Learning of High-Dimensional Neural Network Potentials for Transition Metals. ACS Appl. Mater. Interfaces 2024, 16, 20681–20692. 10.1021/acsami.3c15399. 38593033

[ref14] López-Zorrilla J.; Aretxabaleta X. M.; Manzano H. Exploring the Polymorphism of Dicalcium Silicates Using Transfer Learning Enhanced Machine Learning Atomic Potentials. J. Chem. Theory Comput. 2024, 20, 7682–7690. 10.1021/acs.jctc.4c00479. 39171744

[ref15] Liu X.; Zhang L.; Liu J. Machine learning phase space quantum dynamics approaches. J. Chem. Phys. 2021, 154, 184104 10.1063/5.0046689. 34241027

[ref16] Chanussot L.; et al. Open Catalyst 2020 (OC20) Dataset and Community Challenges. ACS Catal. 2021, 11, 6059–6072. 10.1021/acscatal.0c04525.

[ref17] López-Zorrilla J.; et al. ænet-PyTorch: A GPU-supported implementation for machine learning atomic potentials training. J. Chem. Phys. 2023, 158, 164105 10.1063/5.0146803. 37096855

[ref18] Chen M. S.; Morawietz T.; Mori H.; Markland T. E.; Artrith N. AENET–LAMMPS and AENET–TINKER: Interfaces for accurate and efficient molecular dynamics simulations with machine learning potentials. J. Chem. Phys. 2021, 155, 074801 10.1063/5.0063880. 34418919

[ref19] Behler J. First Principles Neural Network Potentials for Reactive Simulations of Large Molecular and Condensed Systems. Angew. Chem. 2017, 129, 13006–13020. 10.1002/ange.201703114. 28520235

[ref20] Artrith N.; Morawietz T.; Behler J. High-dimensional neural-network potentials for multicomponent systems: Applications to zinc oxide. Phys. Rev. B: Condens. Matter Mater. Phys. 2011, 83, 153101 10.1103/PhysRevB.83.153101.

[ref21] Samanta A. Representing local atomic environment using descriptors based on local correlations. J. Chem. Phys. 2018, 149, 244102 10.1063/1.5055772. 30599737

[ref22] Goscinski A.; Fraux G.; Imbalzano G.; Ceriotti M. The role of feature space in atomistic learning. Mach. Learn.: Sci. Technol. 2021, 2, 025028 10.1088/2632-2153/abdaf7.

[ref23] Cheng H. Feature Representation and Learning In Sparse Representation, Modeling and Learning in Visual Recognition: Theory, Algorithms and Applications 155–181, Springer: London, 2015.

[ref24] Frederic R.; Riad R. Deep Feature selection In Feature and Dimensionality Reduction for Clustering with Deep Learning 131–149, Springer Nature: Switzerland, 2024.

[ref25] Zhang Y.; Xia J.; Jiang B. REANN: A PyTorch-based end-to-end multi-functional deep neural network package for molecular, reactive, and periodic systems. J. Chem. Phys. 2022, 156, 114801 10.1063/5.0080766. 35317591

[ref26] Smith J. S.; Isayev O.; Roitberg A. E. ANI-1: an extensible neural network potential with DFT accuracy at force field computational cost. Chem. Sci. 2017, 8, 3192–3203. 10.1039/C6SC05720A. 28507695 PMC5414547

[ref27] Loshchilov I.; Hutter F. Decoupled Weight Decay Regularization. Published as a conference paper at International Conference on Learning Representations (ICLR) 2019, arXiv:1711.05101v3 (2019), 10.48550/arXiv.1711.05101.

[ref28] Perich M. P.; et al. In Situ Analysis of Gas Dependent Redistribution Kinetics in Bimetallic Au-Pd Nanoparticles. J. Mater. Chem. A 2024, 12, 32760–32774. 10.1039/D4TA03030C. PMC11627011 39659480

[ref29] Cohen J. Statistical Power Analysis for the Behavioral Sciences Second Edition. Lawrence Erlbaum Associates: New York, ISBN 9780203771587 (1998).

