# Supplementary material for: Cost-Effective Strategy of Enhancing Machine Learning Potentials by Transfer Learning from a Multicomponent Data Set on ænet-PyTorch
Source: J Phys Chem C Nanomater Interfaces. 2024 Dec 27;129(1):658–69. doi: 10.1021/acs.jpcc.4c06235 (PMC11726662; doi:10.1021/acs.jpcc.4c06235)
Supplement: Supplementary file 1 — jp4c06235_si_001.pdf [file jp4c06235_si_001.pdf]

Supporting Information

A cost-effective strategy of enhancing machine learning potentials by transfer learning from a multicomponent dataset on ænet-PyTorch

An Niza El Aisnada[a,b], *Kajjana Boonpalit*[b,d], *Robin van der Kruit*[b], *Koen M. Draijer*[b], *Jon Lopez-Zorrilla*[c], *Masahiro Miyauchi*[a], *Akira Yamaguchi*[a,e], *Nongnuch Artrith*[b*]

[a] Department of Materials Science and Engineering, School of Materials and Chemical Technology, Tokyo Institute of Technology, 2−12−1 Ookayama, Meguro-ku, Tokyo 152−8552, Japan

[b] Materials Chemistry and Catalysis, Debye Institute for Nanomaterials Science, Utrecht University, 3584 CG Utrecht, The Netherlands

[c] Physics Department, University of the Basque Country (UPV/EHU), Leioa, Basque Country, Leioa 48940, Spain

[d] School of Information Science and Technology, Vidyasirimedhi Institute of Science and Technology, Rayong 21210, Thailand

[e] Biofunctional Catalyst Research Team, RIKEN Center for Sustainable Resource Science, 2-1 Hirosawa, Wako, Saitama 351-0198, Japan

Keywords: machine learning interatomic potential, artificial neural networks, transfer learning, catalysis, material simulation, ænet-PyTorch

S1. CuAu/H_2O dataset

All *ab initio* molecular dynamics (AIMD) simulations were run for a total time of 10 ps (picoseconds) with a time step of 1 fs to obtain converged trajectories for CuAu nanoparticles with 2, 4, 6, and 8 water (H_2O) molecules. The FHI-aims code[1], developed at the Fritz Haber Institute in Berlin, was used for the AIMD calculations. The generalized gradient approximation (GGA) exchange-correlation functional by Perdew, Burke, and Ernzerhof (PBE)[2] was employed in combination with relativistic corrections at the level of the Atomic ZORA approximation[3], using a time step of 1 fs. The MD runs were conducted with the NVT canonical ensemble using the Bussi-Donadio-Parrinello thermostat at 400 K. Additionally, the method by Tkatchenko and Scheffler[4] was used to correct for the missing van der Waals interactions at this level of DFT. For optimal computational efficiency, AIMD simulations with FHI-aims employed the predefined light basis set (4th order expansion of the Hartree potential, radial integration grids with 302 points in the outer shell, and a tier 1 basis set).

S2. Cu-Au-Pt/O_2 dataset

For the calculations involved in the Pt addition on an Au-terminated 100 CuAu surface and the subsequent O2 dissociation on the Pt-coated surface, DFT calculations with FHI-aims code[1] were utilized. All calculations were performed with the PBE functional[2], atomic ZORA scalar relativistic effects[3] and an SCF convergence of 1E-6 e/a_o^3.

Filling of the Au-terminated surface with Pt atoms followed three different addition schemes: sequentially, as a cluster, or as a monolayer. For each addition step, a two-step geometry optimization was performed with the 'light' and 'tight' basis sets and the Tkatchenko-Scheffler dispersion model[4]. The geometry was optimized with the BFGS algorithm and a convergence of 5E-3 eV/AA on a 12 x 12 x 1 k-grid. One of the obtained Pt-coated surfaces was used for the dissociation investigation of O_2 on the Pt-coated surface using a nudged elastic band (NEB) as implemented in ASE, where the 'light' basis set was used without dispersion correction on a 5 x 5 x 1 k-grid. In an initial run, 5 images were used between the beginning and end minima (O_2 and 2 O on the surface), followed by Climbing Image NEB between the structures around the observed maximum to obtain the activation energy.

S3. Direct application of pre-training MLP model on Cu-Au-Pt/O_2

We calculated the adsorption energies of the Au-terminated CuAu (100) surface during Pt addition, followed by predicting the relative energy during O_2 dissociation on the Pt-coated

surface (DFT at PBE level with FHI-aims package[1], from Robin van der Kurit's Master Thesis 2023[5]). The average predictions for the MLPs during addition and dissociation are shown in Figure S1 (a) and (b). The range of predictions for the MLPs during addition is shown in Figure S1 (a), with two of the structures with 8 Pt atoms shown in Figure S1 (b) and (c). The MLP adsorption energy increases with additional Pt atoms, whereas the DFT results show a decrease in adsorption energy. An additional comparison was performed between the DFT and MLP energies for the dissociation of O_2 with nudged elastic band (NEB) calculations. The relative DFT energy and errors per MLP model for each NEB image is shown in Table S1. The MLP models start with negative error in the energy before inverting to a positive error, showing that the MLP models do not capture the activation energy nor overall energy change. If the NEB data is plotted, the MLP models do not show any trends besides a roughly horizontal line.

In neither of the above-described cases do the MLP results follow the same trends as the DFT results, indicating that the models may be lacking in some aspects. Based on the O_2 dissociation results, it is possible that these discrepancies arise from the calculations being outside the training domain, causing the model to struggle with these structures. Another observation from the Pt addition results, when comparing the individual model outcomes, is that the models do not produce similar plot shapes in this scenario. This may indicate insufficient training data in the random selection or a difference emerging during training. The Pt-coated surfaces used in the dissociation part of this task do not have Pt atoms following the existing CuAu lattice. Instead, the surface features regions with higher (closer together) and lower Pt concentrations, resulting in a distorted minimum structure. However, this does not explain the poor performance for Pt addition, as the highly regular Pt-coated structure is part of the dataset. It is thus required to further improve the MLP models to solve the discrepancies between the MLP and DFT results.

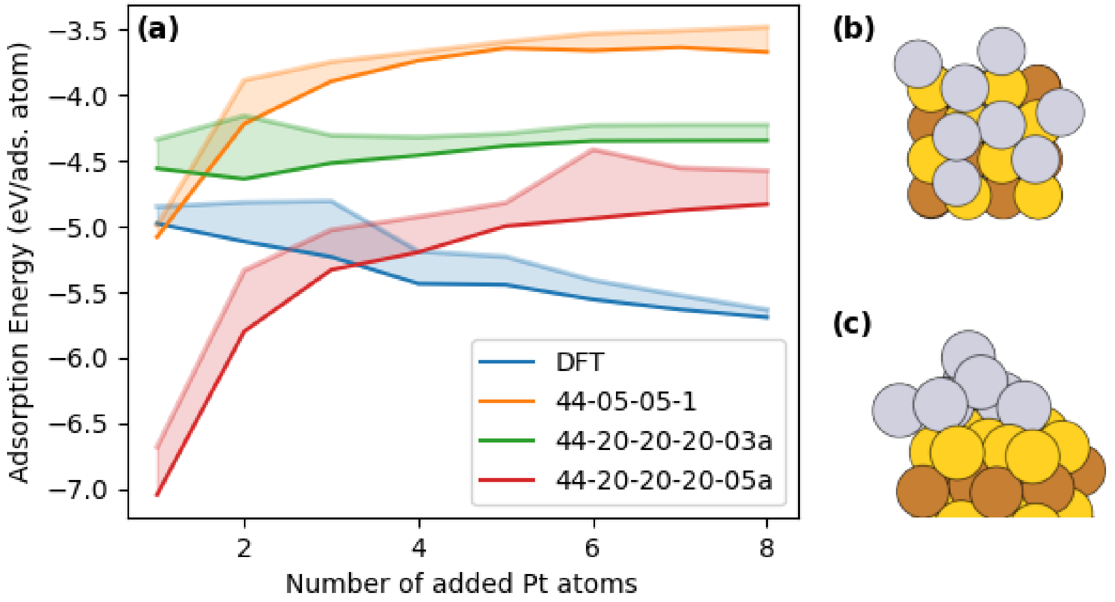

Figure S1. (a) Comparison of three MLPs ænet-Pytorch models to the DFT results for the sequential addition of Pt on an Au-terminated (100) CuAu surface where the top two layers are allowed to move. For each energy model, the lowest energies are shown with a solid line, while the shaded area represents the range of energies for a given number of Pt atoms. Two obtained structures for the addition of 8 Pt atoms **(b)** sequentially and **(c)** as a cluster.

Table S1. Relative DFT energy in eV for each NEB image and the error per each machine learning potential (MLP) model for the dissociation of O_2 on a Pt coated Au-terminated CuAu(100) surface.

**NEB index**	**Rel. Energy (eV)**	**Error in Relative Energy (eV) to DFT**		
		**44-05-05-1-03a**	**44-20-20-20-03a**	**44-20-20-20-05a**
**0**	0	0	0	0
**1**	0.19	-0.32	-0.28	-0.26
**2**	0.22	-0.15	-0.50	-0.26
**3**	0.18	-0.22	-0.42	-0.15
**4**	0.12	-0.39	-0.45	-0.35
**5**	-0.04	-0.42	-0.38	-0.46
**6**	-0.53	-0.03	0.25	0.30
**7**	-1.23	0.74	1.13	1.14
**8**	-1.37	0.64	1.34	0.89

S3. Direct application of pre-training MLP model on H_2/O_2 adsorption on Pd-Au alloy

We calculated the adsorption energies of H_2 and O_2 on a Pd-Au alloy surface. The bottom five layers are a random mixture of Pd and Au in a 0.45:1 ratio (frozen during calculation), and the surface layer is made up of 16 atoms, which vary in composition of 0:1 Pd:Au to 1:0 Pd:Au. The adsorption energy was evaluated for the adsorbate for the same on-top adsorption site in each model. This adsorption site was where the Pd atoms were introduced into the full Au surface layer. Computational details for the DFT calculation can be found in Reference [6]. We found that the 44-05-05-1-03a and 44-20-20-20-03a potentials have an absolute error in the O_2 adsorption energy smaller than 1 eV with the surface models where the Pd fraction is below 0.5 (Figure S2a). Interestingly, the error of all three potentials is smaller for a surface with a 1:0.07 Pd:Au ratio compared to a full Pd surface. Although the worst performing potential for the full O_2 adsorption dataset, the 44-20-20-20-05a potential has an error of only 0.04 eV for the full Au surface. The 44-05-05-1-03a potential has a mean absolute error (MAE) of 0.61 eV for the O_2 adsorption dataset, which is the lowest of the three tested machine-learned potentials (Table S2). As the adsorption energies are in the range of 0.76-2.26 eV, this MAE is too large for the machine learning potentials to be reliable for estimating the energy of O_2 adsorption on Pd-Au alloy surfaces.

In the case of H_2 adsorption energies, the 44-20-20-20-03a and 44-20-20-20-05a potentials perform better than the 44-05-05-1-03a with the low Pd surface fractions, with an absolute error below 1 eV (Figure S2b). In the case of a pure Au surface, the 44-20-20-20-03a potential performs best with an absolute error of 0.02 eV. However, for the estimation of the H_2 adsorption on the Pd-rich surfaces, the 44-20-20-20-03a potential has absolute errors above 3 eV. In this regime, the 44-20-20-20-05a has an absolute error for the H_2 adsorption energy on the pure Pd surface of only 0.26 eV. From the tested potentials, the 44-20-20-20-05a potential has the lowest MAE of 0.51 eV for the H_2 adsorption dataset (Table S2). Similar to the O_2 adsorption energy dataset, the MAEs of the machine learning potentials make them unreliable for the prediction of H_2 adsorption energies on Pd-Au alloy surfaces, which had a range of 0.06-1.54 eV.

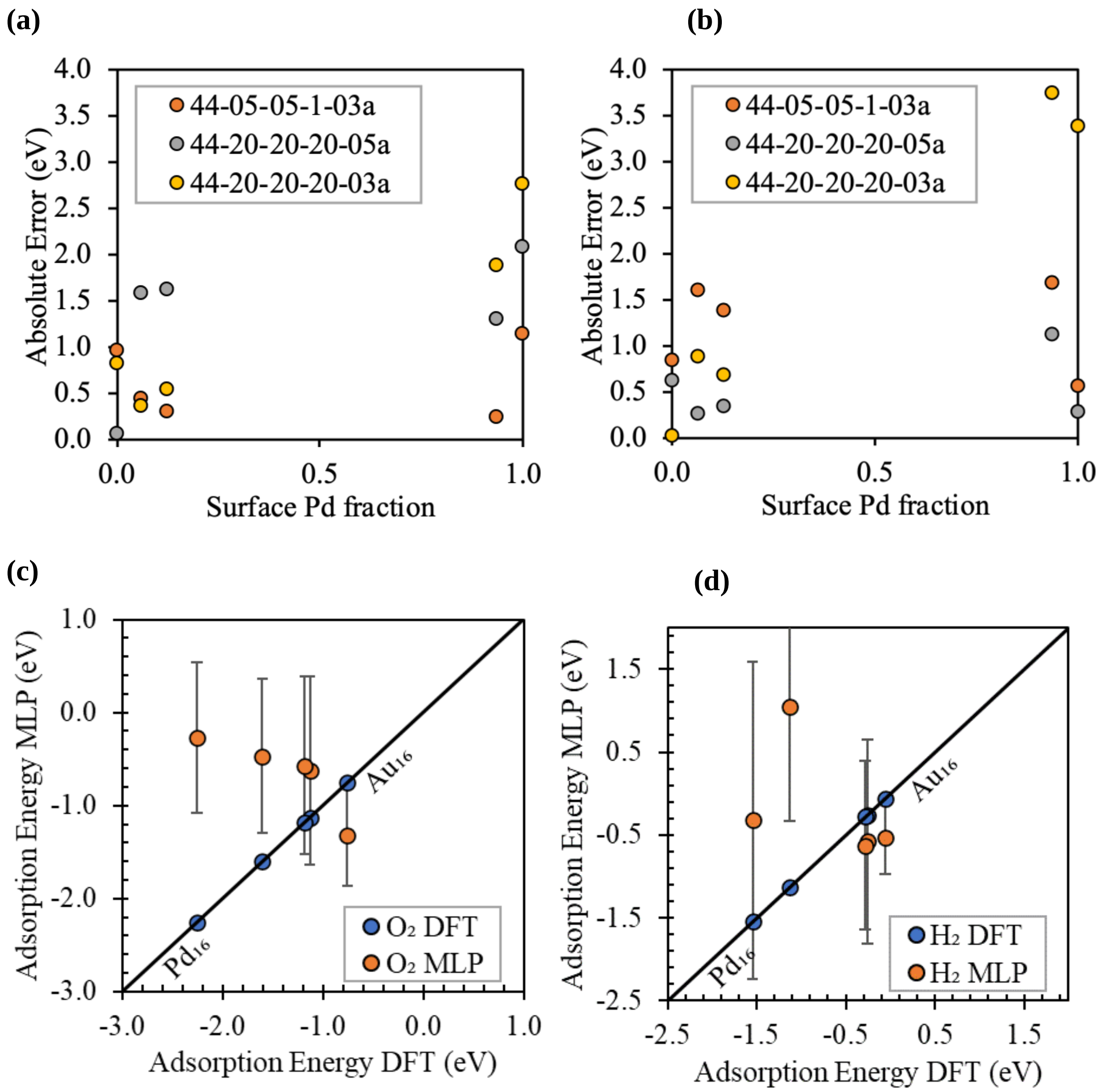

Figure S2. (a) Absolute error between the OC20 machine learning potentials and PBE+TS DFT for the calculated adsorption energy of molecular O_2 on random alloy Pd-Au (111) surfaces. (b) Absolute error between the OC20 machine learning potentials and PBE+TS DFT for the calculated adsorption energy of H_2 on random alloy Pd-Au (111) surfaces. (c-d) Average predictions from 3 MLPs for the adsorption energy of O_2 and H_2 on random alloy Pd-Au (111) surfaces.

Table S2. Mean Average Error (MAE) in eV per machine learning potential (MLP) for O_2/H_2 adsorption on Pd-Au alloy.

MLP	MAE (eV) O_2 adsorption	MAE (eV) H_2 adsorption
**44-05-05-1-03a**	0.61	1.20
**44-20-20-20-05a**	1.32	0.51
**44-20-20-20-03a**	1.26	1.73

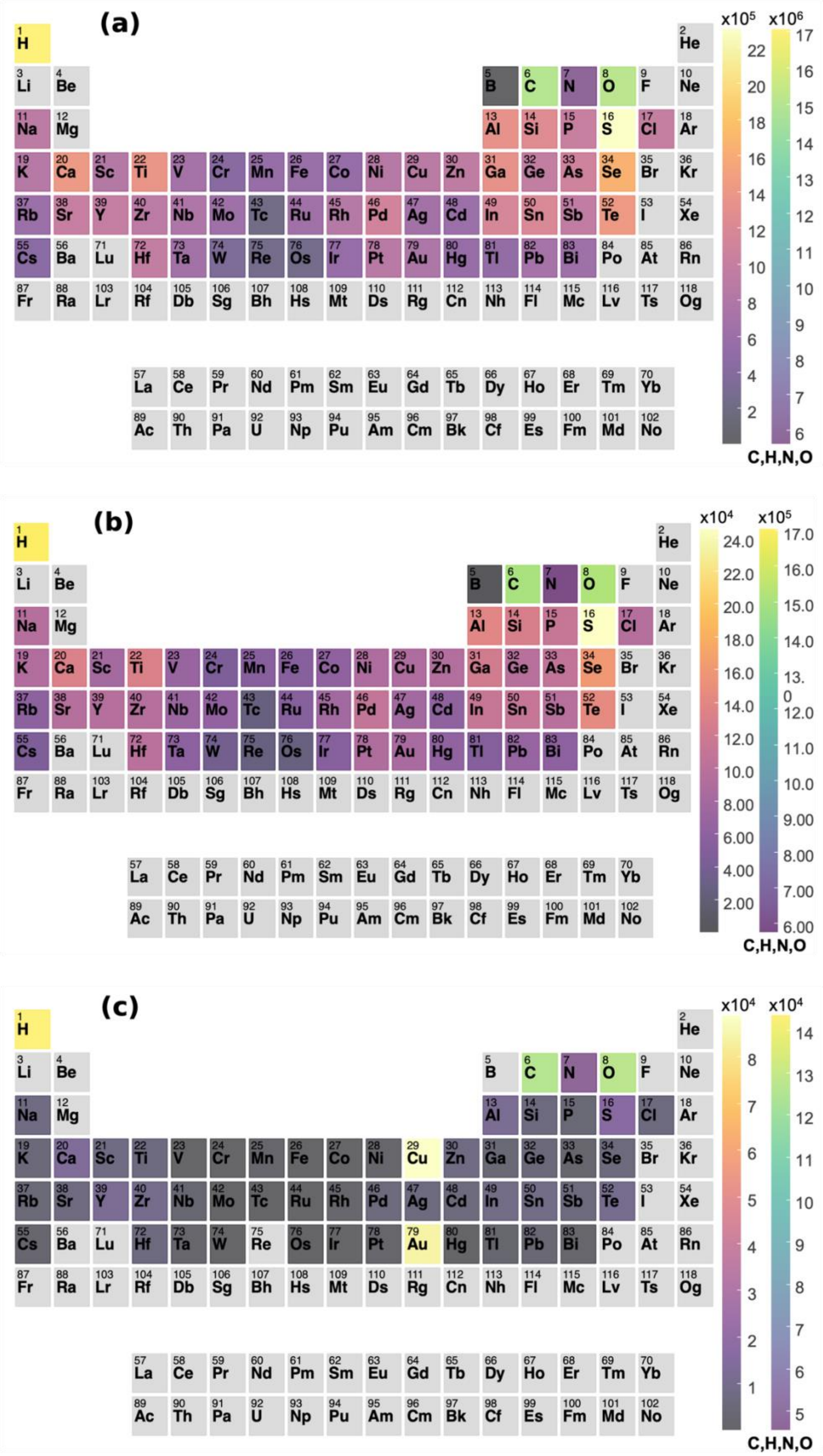

(a)
x10^5
x10^6
C,H,N,O
(b)
x10^4
x10^5
C,H,N,O
(c)
x10^4
x10^4
C,H,N,O

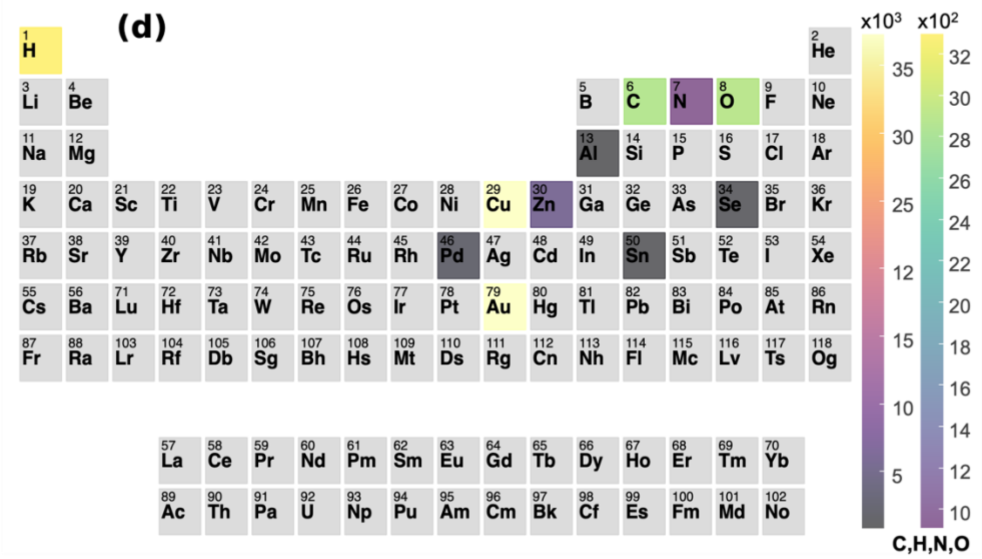

Figure S3. Elements distribution of the **(a)** OC20 database, **(b)** Random subset, **(c)** CuAu-54 subset, and **(d)** CuAu-11 subset.

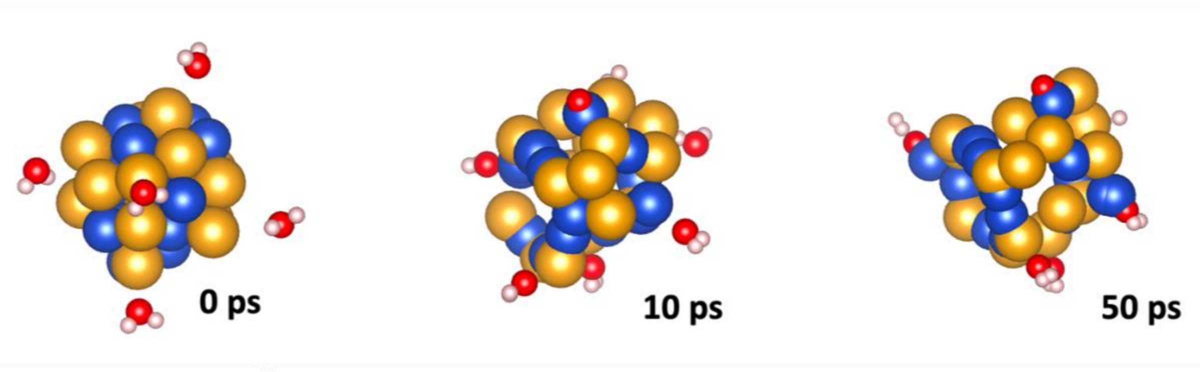

Figure S4. MD snapshots of CuAu/6H_2O at 0, 10, and 50 ps, performed by OC20 pre-trained model directly. The structure is distorted by inaccurate MLP due to the absence of metal cluster data in the OC20.

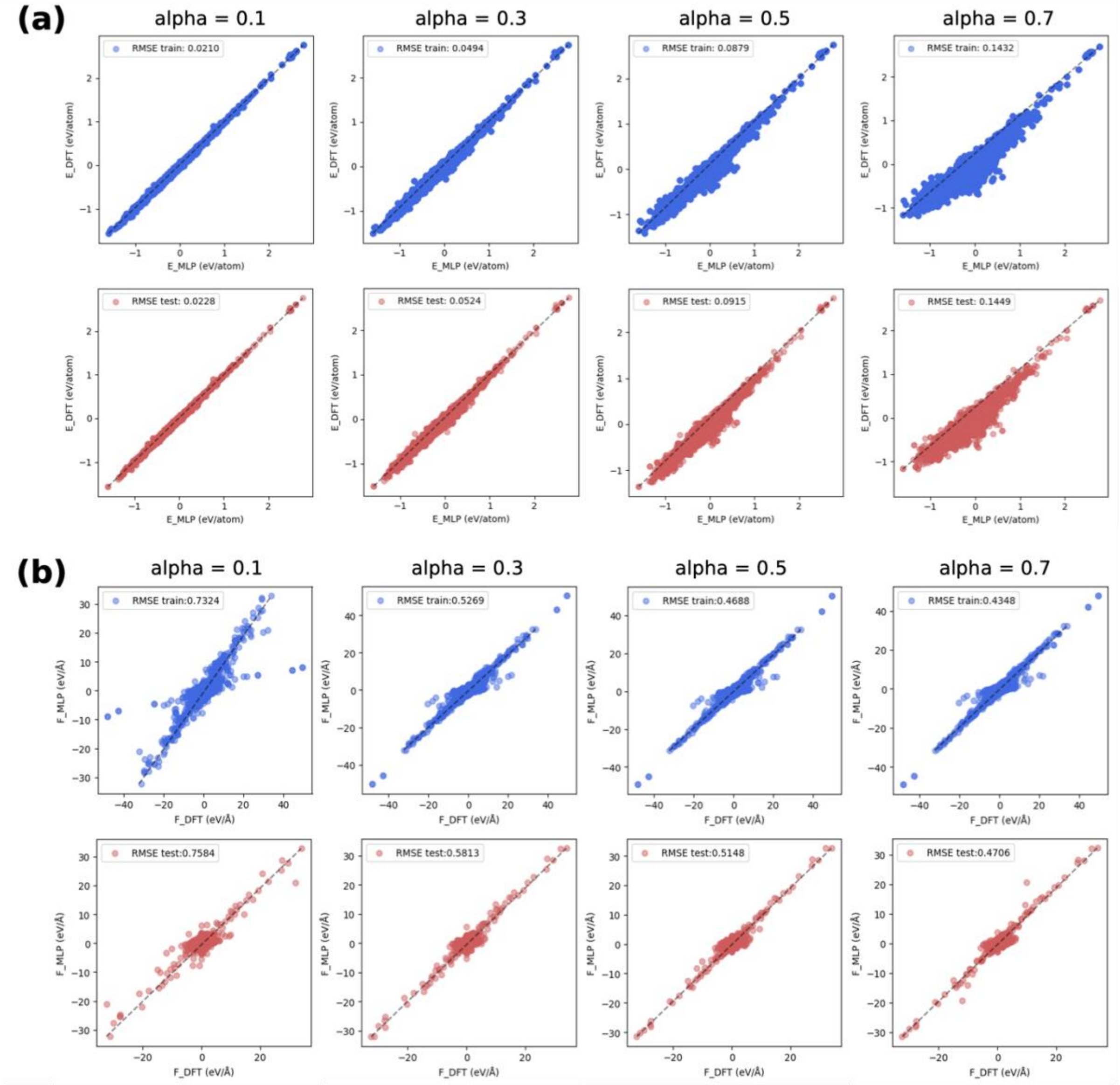

Figure S5. MLP accuracy according to the variation of alpha value (α = 0.1, 0.3, 0.5, 0.7) trained on the OC20 subset. The blue dots indicate the training data, and the red dots indicate test data during the training process.

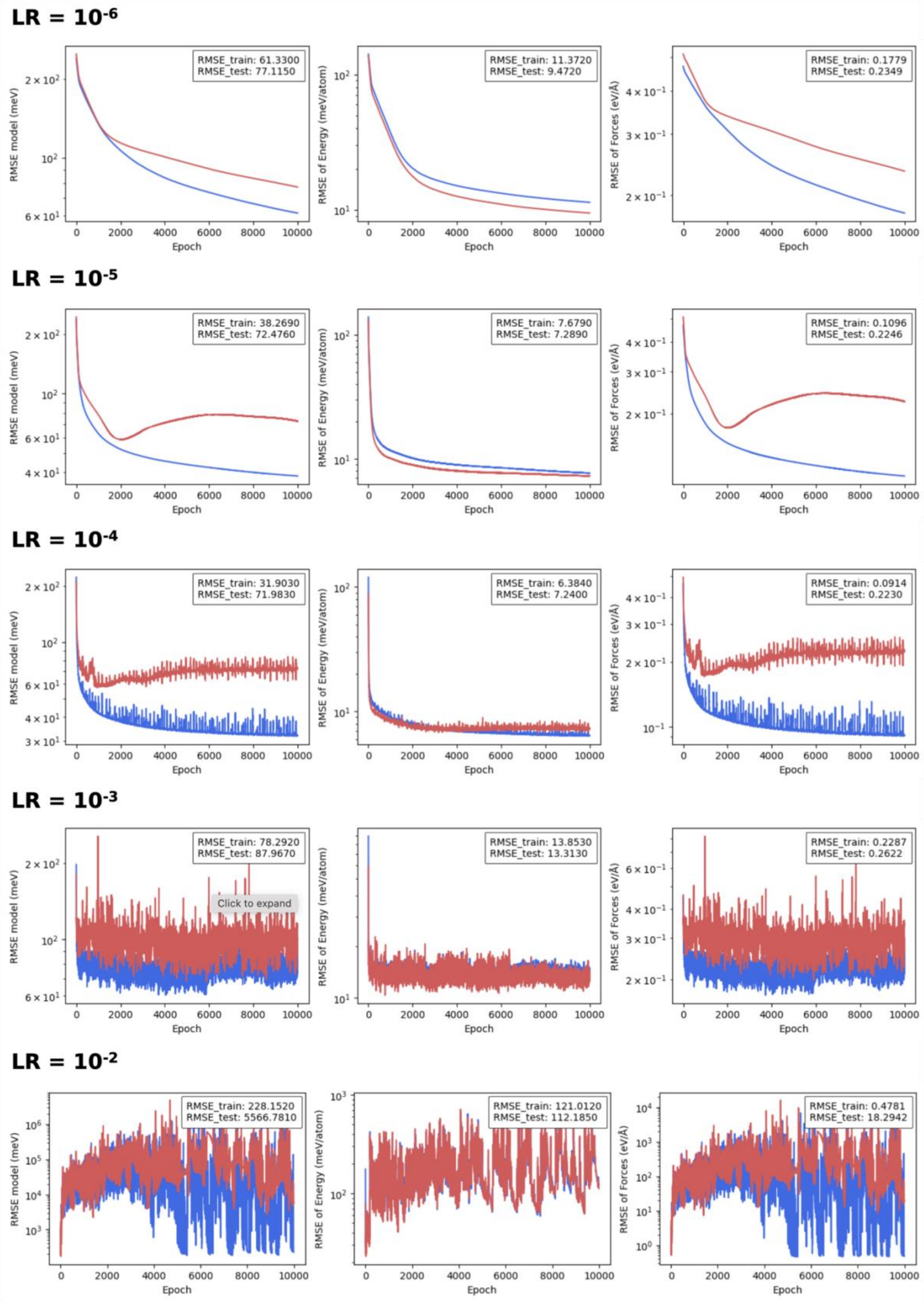

Figure S6. The overall prediction convergence (including energy and force) during the MLP training process for the OC20 subset using the Adam optimizer with LR from 10^{-6} to 10^{-2}.

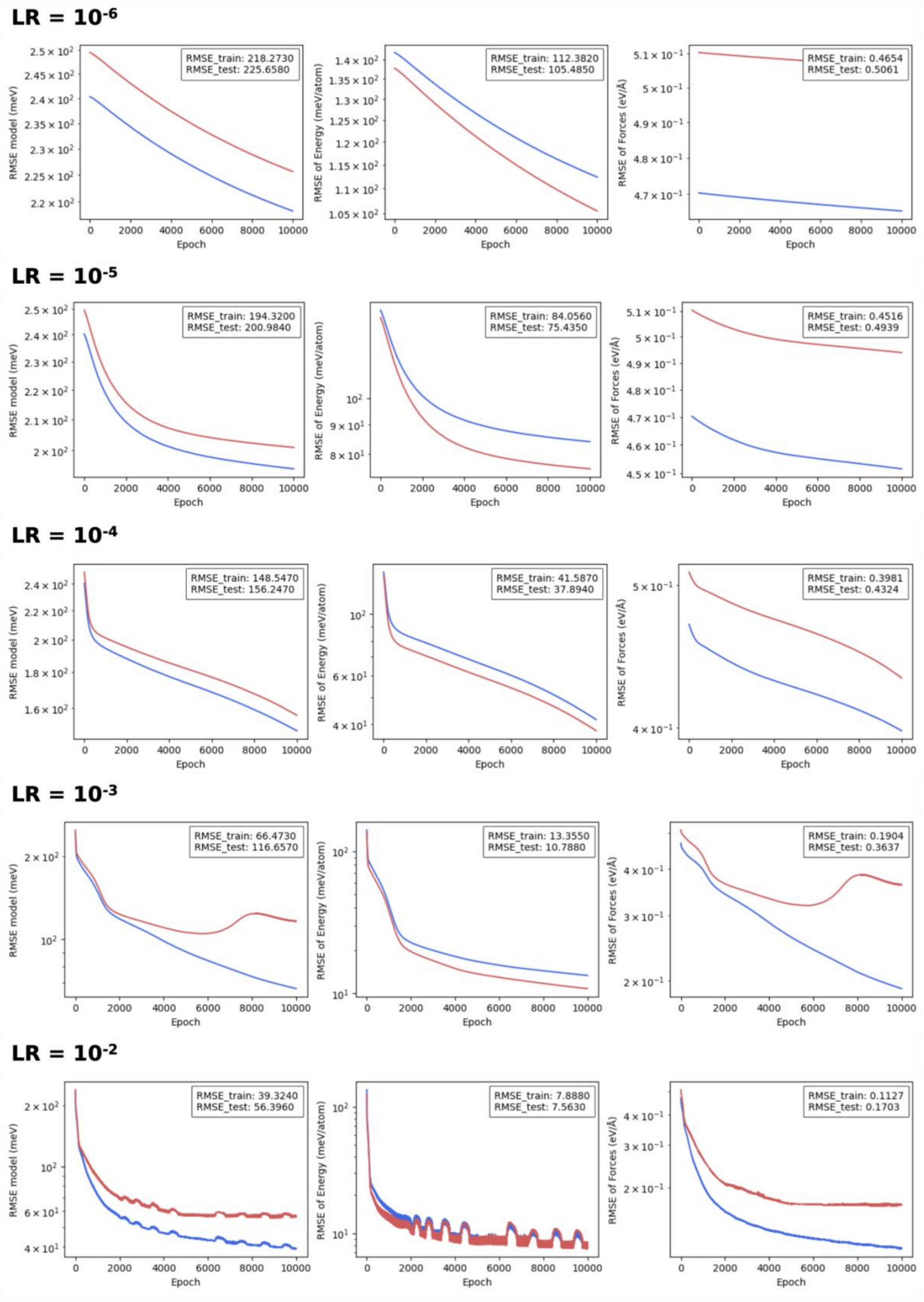

Figure S7. The overall prediction convergence (including energy and force) during the MLP training process for the OC20 subset using the Adadelta optimizer with LR from 10^{-6} to 10^{-2}.

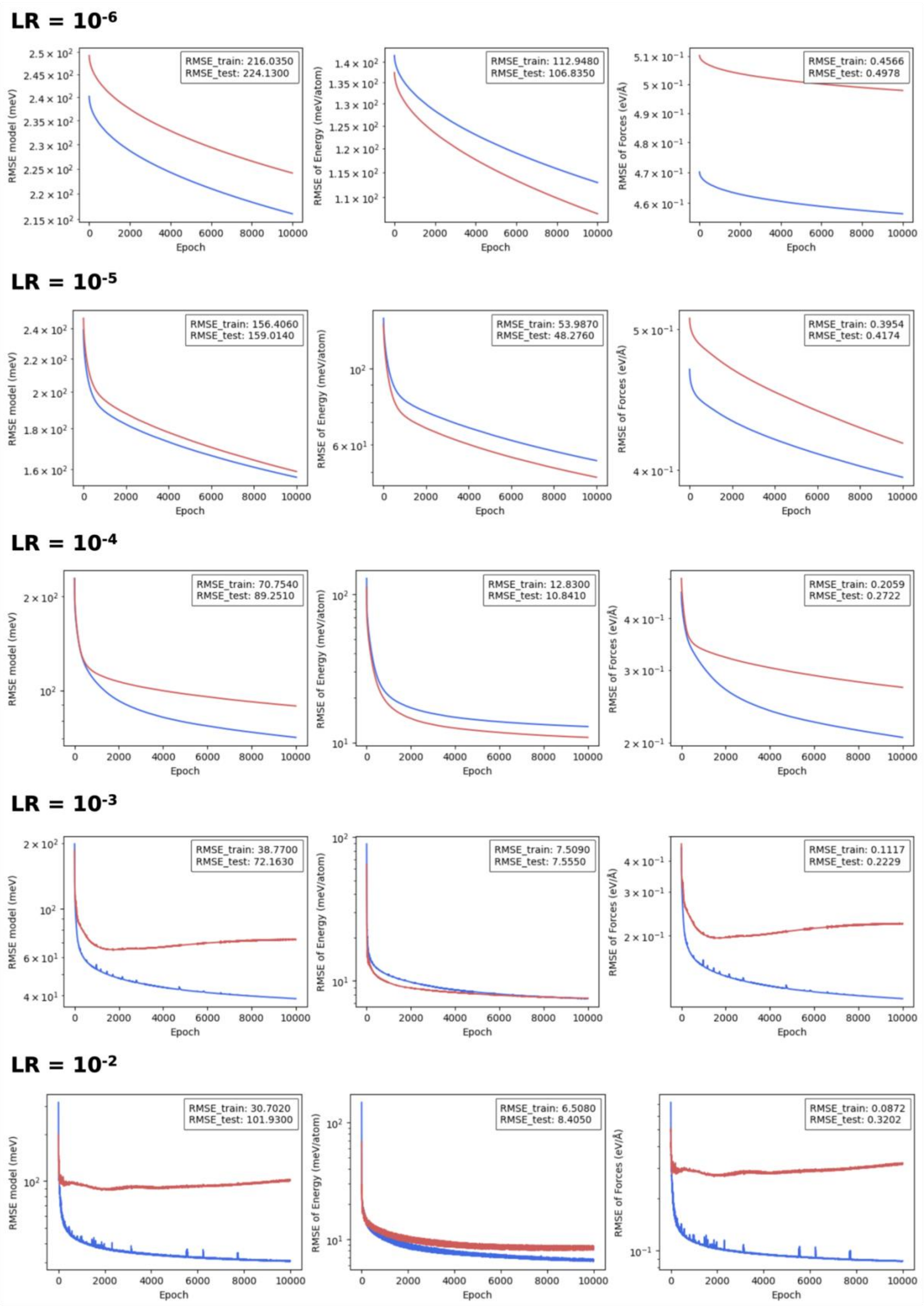

Figure S8. The overall prediction convergence (including energy and force) during the MLP training process for the OC20 subset using the Adagrad optimizer with LR from 10^{-6} to 10^{-2}.

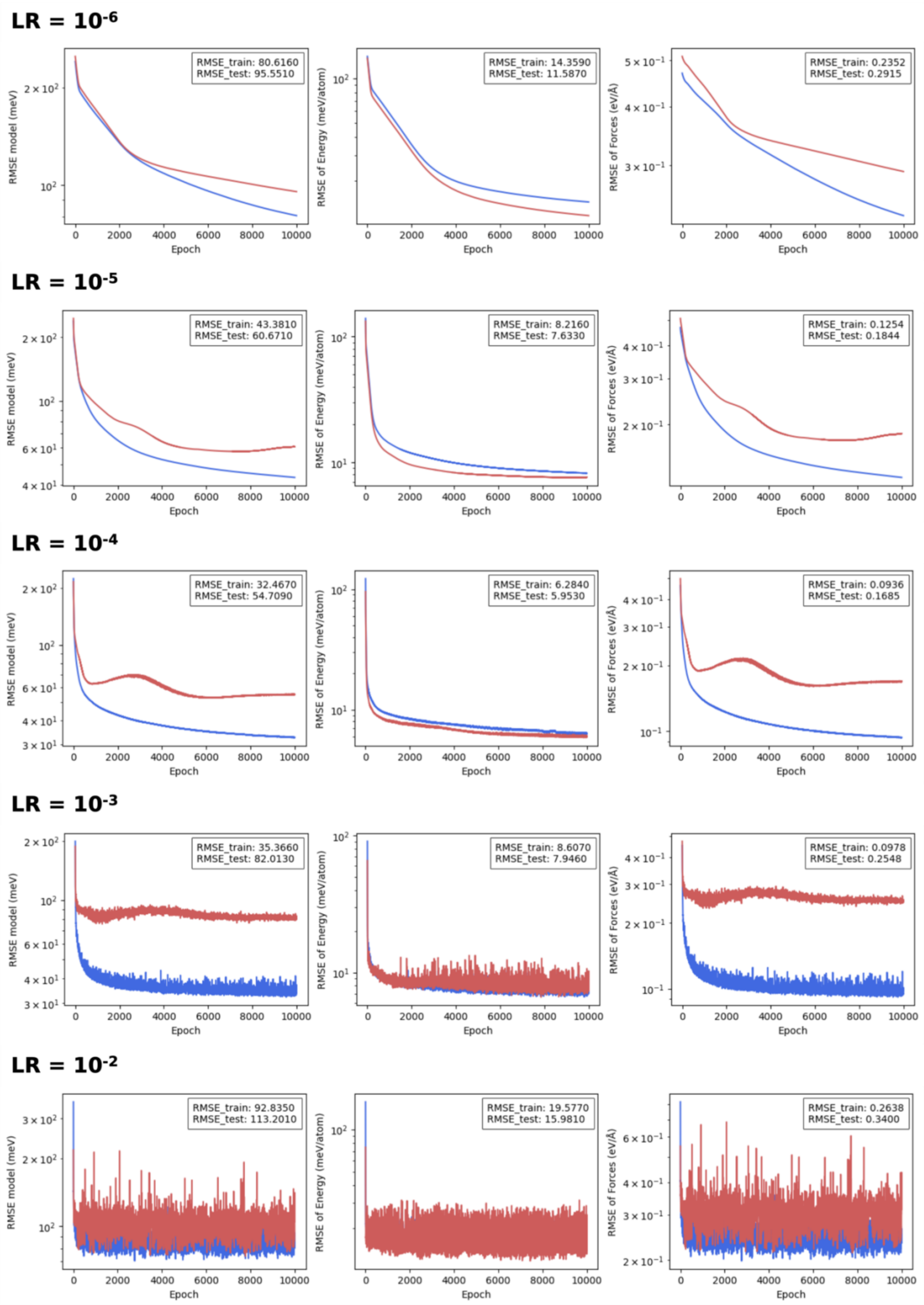

Figure S9. The overall prediction convergence (including energy and force) during the MLP training process for the OC20 subset using the Adamax optimizer with LR from 10^{-6} to 10^{-2}.

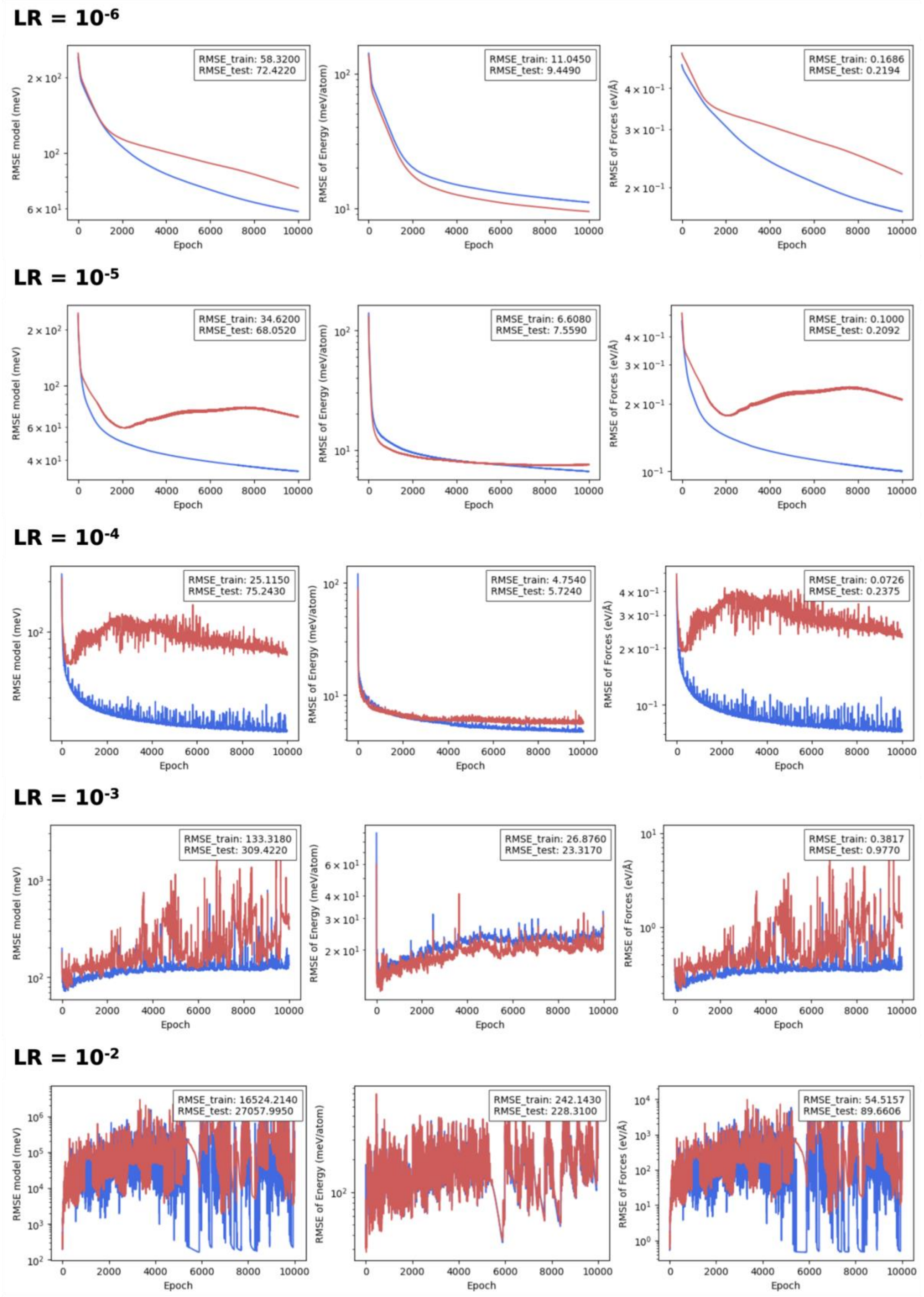

Figure S10. The overall prediction convergence (including energy and force) during the MLP training process for the OC20 subset using the Adamw optimizer with LR from 10^{-6} to 10^{-2}.

Table S3. The RMSE values of MLP models for the CuAu/H_2O dataset used to plot Figure 9.

Model	RMSE (E_train) (meV)	RMSE (E_test) (meV)	RMSE (F_train) (meV/Å)	RMSE (F_test) (meV/Å)
Scratch	63.89	64.18	112.13	112.15
TF-Random	63.86	64.10	103.18	103.49
TF-54	63.85	64.05	103.15	103.48
TF-11	63.82	64.03	100.72	100.75

REFERENCES

1. Blum, V. *et al.* Ab initio molecular simulations with numeric atom-centered orbitals. *Comput Phys Commun* **180**, 2175–2196 (2009).
2. Perdew, J. P., Burke, K. & Ernzerhof, M. Generalized Gradient Approximation Made Simple. *Phys Rev Lett* **77**, 3865–3868 (1996).
3. van Lenthe, E., Baerends, E. J. & Snijders, J. G. Relativistic total energy using regular approximations. *J Chem Phys* **101**, 9783–9792 (1994).
4. Tkatchenko, A. & Scheffler, M. Accurate Molecular Van Der Waals Interactions from Ground-State Electron Density and Free-Atom Reference Data. *Phys Rev Lett* **102**, 73005 1–4 (2009).
5. Robin van der Kruit. Master Thesis. (Utrecht University, The Netherlands, 2023).
6. Perxés Perich, M. *et al.* In situ analysis of gas dependent redistribution kinetics in bimetallic Au-Pd nanoparticles. *J. Mater. Chem. A.* **12**, 32760–32774 (2024).
